# Plasmid diversity and phylogenetic consistency in the Lyme disease agent *Borrelia burgdorferi*

**DOI:** 10.1186/s12864-017-3553-5

**Published:** 2017-02-15

**Authors:** Sherwood R. Casjens, Eddie B. Gilcrease, Marija Vujadinovic, Emmanuel F. Mongodin, Benjamin J. Luft, Steven E. Schutzer, Claire M. Fraser, Wei-Gang Qiu

**Affiliations:** 1 Division of Microbiology and Immunology, Pathology Department and Biology Department, University of Utah School of Medicine, Room 2200 K Emma Eccles Jones Medical Research Building, 15 North Medical Drive East, Salt Lake City, UT 84112 USA; 2 Biology Department, University of Utah, Salt Lake City, UT USA; 3 Institute for Genome Sciences, University of Maryland BioPark, Baltimore, MD USA; 4 Department of Medicine, Health Science Center, Stony Brook University, Stony Brook, NY USA; 5 Department of Medicine, New Jersey Medical School, Rutgers, the State University of New Jersey, Newark, NJ 07103 USA; 6 Department of Biology, The Graduate Center, City University of New York City, New York, NY USA; 7 Department of Biological Sciences and Center for Translational and Basic Research, Hunter College of the City University of New York City, New York, NY USA; 8 Present Address: Janssen Disease and Vaccines, Pharmaceutical Companies of Johnson and Johnson, Leiden, The Netherlands

**Keywords:** *B. burgdorferi*, Plasmid, Linear plasmid, Genome rearrangement

## Abstract

**Background:**

Bacteria from the genus *Borrelia* are known to harbor numerous linear and circular plasmids. We report here a comparative analysis of the nucleotide sequences of 236 plasmids present in fourteen independent isolates of the Lyme disease agent *B. burgdorferi*.

**Results:**

We have sequenced the genomes of 14 *B. burgdorferi* sensu stricto isolates that carry a total of 236 plasmids. These individual isolates carry between seven and 23 plasmids. Their chromosomes, the cp26 and cp32 circular plasmids, as well as the lp54 linear plasmid, are quite evolutionarily stable; however, the remaining plasmids have undergone numerous non-homologous and often duplicative recombination events. We identify 32 different putative plasmid compatibility types among the 236 plasmids, of which 15 are (usually) circular and 17 are linear. Because of past rearrangements, any given gene, even though it might be universally present in these isolates, is often found on different linear plasmid compatibility types in different isolates. For example, the *arp* gene and the *vls* cassette region are present on plasmids of four and five different compatibility types, respectively, in different isolates. A majority of the plasmid types have more than one organizationally different subtype, and the number of such variants ranges from one to eight among the 18 linear plasmid types. In spite of this substantial organizational diversity, the plasmids are not so variable that every isolate has a novel version of every plasmid *(i.e.,* there appears to be a limited number of extant plasmid subtypes).

**Conclusions:**

Although there have been many past recombination events, both homologous and nonhomologous, among the plasmids, particular organizational variants of these plasmids correlate with particular chromosomal genotypes, suggesting that there has not been rapid horizontal transfer of whole linear plasmids among *B. burgdorferi* lineages. We argue that plasmid rearrangements are essentially non-revertable and are present at a frequency of only about 0.65% that of single nucleotide changes, making rearrangement-derived novel junctions (mosaic boundaries) ideal phylogenetic markers in the study of *B. burgdorferi* population structure and plasmid evolution and exchange.

**Electronic supplementary material:**

The online version of this article (doi:10.1186/s12864-017-3553-5) contains supplementary material, which is available to authorized users.

## Background

Members of the spirochete genus *Borrelia* in both the relapsing fever and Lyme disease agent clades have been found to carry large numbers of linear and circular plasmids that range from 5 to over 200 kbp in size. These linear plasmids were first observed as DNA bands in electrophoresis gels by Barbour [1, 2], and both linear and circular plasmids have subsequently been shown to be universally present in *Borrelia* isolates (e. g. [2–14]). These plasmids have a number of unusual and interesting features. (i) The linear plasmids have covalently-closed hairpin ends [1, 15, 16]. (ii) The plasmids encode many N-terminally lipidated proteins, many of which are targeted to the outer surface of the bacteria (e. g., [17]). These proteins are important in interactions between the bacteria and their hosts and are potential vaccine and detection targets. (iii) Some of the linear plasmids have an unusually low (for bacteria) density of protein coding genes and harbor an unusually large number of pseudogenes [17–19]. (iv) A large number of paralogous gene families and paralogous intergenic sequences are present on the plasmids [17]. (v) The previous two observations suggest a tumultuous history of (often duplicative) rearrangements among the plasmids followed by decay of broken and redundant genes [17–19]. (vi) Most of the plasmids are quite easily lost with growth in culture, which can make maintenence of fully virulent strains in the laboratory difficult [20–22]. (vii) Among the plasmids, only cp26 is known to be required for growth in culture [23–25]. (viii) Up to nine and probably as many as 12 different paralogous versions of the cp32 family of circular plasmids can exist in the same cell [26, 27]. (ix) A number of the plasmids appear to be prophages or prophage-related [17, 28]. (x) Only a few percent of the linear plasmid genes encode proteins with homology to known proteins outside of the *Borrelia* genus, and these include proteins related to previously known plasmid partitioning and maintenance proteins, small molecule transporters, DNA restriction-modification systems, as well as nucleotide and DNA metabolism enzymes (reviewed in [29]). And finally, (xi) several of the plasmids have been shown to carry genes that are important in mouse and/or tick infection [25, 30–41].

Electrophoretic linear plasmid DNA band patterns of *B. burgdorferi* isolates are extremely variable, and a given gene can reside on different sized linear plasmids in different isolates (below, “*B. burgdorferi*” will refer specifically to the *B. burgdorferi* sensu stricto group of isolates). For example, DNA from genes *b31_k19* through *b31_k22* on *B. burgdorferi* type strain B31’s lp36 plasmid hybridized to plasmids across a 24–38 kbp range in Palmer et al.’s [8] analysis of a panel of 15 North American *B. burgdorferi* isolates, and this was confirmed by comparison of the B31 and 297 lp36 nucleotide sequences when they were determined [19]. On the other hand, several of the *B. burgdorferi* plasmids, most notably the circular cp26 and linear lp54, are present and quite similar in all isolates that have been analyzed [7, 14, 17, 19]. Finally, all *B. burgdorferi* isolates that have been examined carry multiple cp32 plasmids; these are circular plasmids, usually between 29 and 33 kbp in length, that are different, but yet are largely homologous throughout their lengths [17, 26, 42]. The cp32 plasmids are almost certainly prophages [28, 43].

The complete plasmid content of a *Borrelia* isolate is difficult to determine by gel electrophoresis because large DNA circles are poorly resolved and because the multiple linear plasmids often have very similar sizes. To date, the complete plasmid complement has been analyzed for only four *B. burgdorferi* isolates, B31, N40, JD1 and 297, which are known to harbor 23, 17, 20 and 20 different plasmids, respectively [17, 19]. Our previous analysis of these four genomes identified 29 possible “compatibility types,” suggesting that a rather large menu of such types may be present in *B. burgdorferi* [19, 29, 44]. We note that plasmid compatibility is not well understood in *Borrelia*, and this issue is discussed in more detail below. In order to understand the plasmid diversity within and among the different Lyme agent *Borrelia* species, we determined the complete genome sequence of ten additional genetically diverse *B. burgdorferi* isolates, during which we obtained high quality sequences of nearly all of their plasmids [17, 45, 46]. Here, we describe the plasmids present in these *B. burgdorferi* isolates and compare their genetic contents and organizational features.

## Results and discussion

### *B. burgdorferi* genome sequences reveal new plasmid types

#### B. burgdorferi *sensu stricto plasmid putative “compatibility types”*

In addition to those of strains B31, N40, 297 and JD1 which we described previously [17, 19], this study describes and compares the plasmids present in the ten previously unanalyzed *B. burgdorferi* isolates 64b, 72a, 94a, 118a, 156a, 29805, Bol26, CA-11.2A, WI91-23 and ZS7, whose complete genome sequences were determined by Sanger dideoxynucleotide sequencing [47] to an average depth of 8-fold as previously described (available in the GenBank database with plasmid accession numbers and BioProjects listed in references [46, 48]). All the plasmid sequences, with the following few exceptions, were closed and finished to the J. Craig Venter Institute’s “gold standard” of sequence determination: strain 94a plasmid lp28-8 and 156a plasmid lp21 were not closed due to difficulties in assembling long repeat tracts; one WI91-23 cp9 sequence was not closed because of assembly difficulties due to regions of high similarity in another cp9 present in this strain; and Bol26 cp32-4, cp32-10 and cp32-11 sequences were not closed due to their high similarity and to a lack of funds (see NCBI Bioprojects 20999, 19835, 28627 and 19837, respectively, for the sequences of the contigs from these plasmids).

**Fig. 1:**
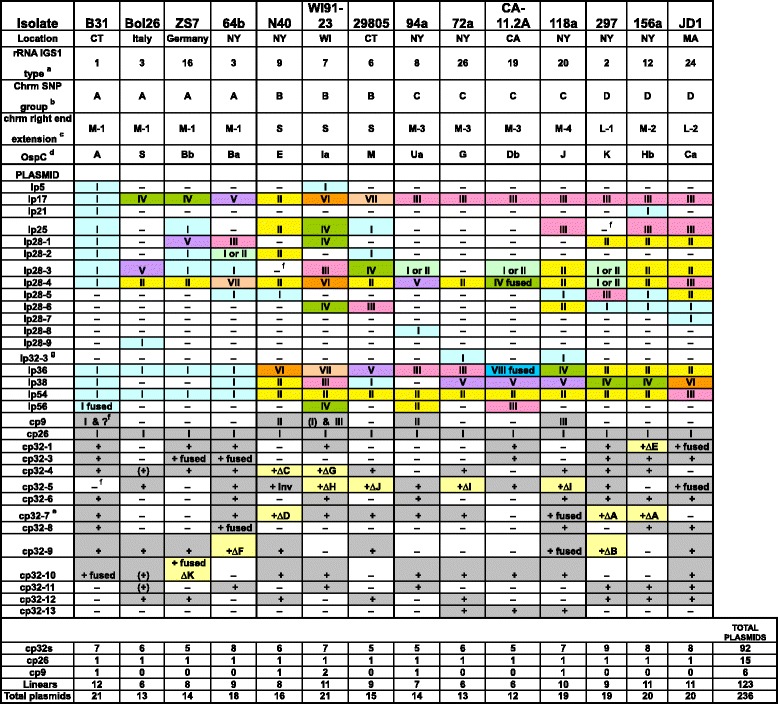
Sequenced *B. burgdorferi* sensu stricto plasmids. The 14 completely sequenced genomes shown as columns where shaded cells indicate the presence of a plasmid. For the linear plasmids, different cell colors and Roman numerals indicate subtypes; similar colors and numerals in different plasmids (lines in table) do not imply any relationship. For the circular plasmids light yellow shading marks the cp32 plasmids with large deletions, and Roman numerals denote the subtype of the cp9 and cp26 plasmids. No subtypes were defined for the cp32s, so a “ + ” indicates a cp32 of that compatibility group is present. “I or II” denotes plasmids in which unsequenced terminal regions preclude discrimination between subtype I and II; “fused” indicates that the two so indicated plasmids in affected strains appear to be covalently joined; “∆”, indicates the presence of a large deletion relative to other cp32s, and the associated letters indicate different deletion endpoints (see Additional file 1: Fig. S4 below); “inv” indicates a large inversion is present relative to the other cp32s; and parentheses (…) enclose the one cp9 and three cp32 plasmids whose sequences were not closed. (a) rRNA IGS1 (intergenic spacer number one) nomenclature according to Travinsky et al. [88]. (b) Related chromosomal SNP (single nucleotide polymorphism) groups according to Mongodin et al. [48]. (c) Defined in text and Fig. 6a below. (d) OspC type nomenclature of Barbour and Travinsky [79]. (e) Previously named cp32-2 AND cp32-7 have the same compatibility; we use cp32-7 to represent this group. (f) These plasmids are known to have been present in the isolate before sequencing, but were lost in the culture whose DNA was used for sequencing. (g) The lp32-3 plasmid likely has the same compatibility type as cp32-3 (see text)

The members of this panel of 14 *B. burgdorferi* isolates are from southern New England (strain 29805, Connecticut; JD1 Massachusetts), southeast New York (strains 64b, 72a, 94a, 118a, 156a, 297, B31 and N40), Wisconsin (strain WI91-23), California (CA-11.2A), Italy (strain Bol26) and Germany (strain ZS7). They include human, bird and tick isolates, and represent a diverse set of rRNA/OspC types (Fig. 1). These 14 genomes contain 236 plasmids of which 113 are circular and 123 are linear. The individual strains each carry between 6 and 11 circular plasmids and between 6 and 12 linear plasmids, with an average 16.9 total plasmids per isolate. We note that the genome sequences of the three strains that have been studied in the most detail and used most frequently in the laboratory, B31, 297 and N40, appear to have lost two (cp9-2 and cp32-5), one (lp25) and one (lp28-3) plasmid(s), respectively, between their original isolation and genome sequence determination [26, 30, 49–51]. Thus, even though cultures were chosen for sequencing that had been passaged a low number of times in the laboratory, the plasmids sequenced in each strain represent a *minimum* number for the plasmids that might have originally been present. We also note that since the closed hairpin end-containing fragments of the linear plasmids are not cloned into plasmid-based DNA sequencing libraries, an unknown amount of sequence is missing from the termini of most of the linear plasmids. Comparison of the terminal sequences with measured terminal restriction fragment lengths (data not shown), indicates that in most cases the missing sequence is very likely one kbp or less (and Additional file 1: Table S1 in [19], see also ref. [52]).

The *B. burgdorferi* plasmids are named according to their putative compatibility type, which is thought to be determined at least in part by the sequence type of the *Borrelia* PFam32 protein that the plasmid encodes [29]. A large majority of the *Borrelia* plasmids encode a PFam32 protein, but the two smallest types - the circular cp9 and linear lp5 plasmids - do not. The PFam32 protein family is homologous to the ParA proteins of other plasmid partitioning systems [53], and although their role in *Borrelia* plasmid maintenence and compatibility is not yet understood [30, 40, 54–57], the 14 *B. burgdorferi* genome sequences and eight genome sequences of other *B. burgdorferi* sensu lato (Lyme disease agent clade) species that include full plasmid sequences [46, 58–60] contain a total of 345 plasmids, and we find that *no isolate* harbors two or more plasmids with the same PFam32 “sequence type” ([17, 19, 29, 42] and the analysis in this report; see Fig. 1). This strong correlation does not prove, but does lend support to the notion that the PFam32 proteins are involved in conferring plasmid compatibility in spite of the fact that they are not always essential. *Borrelia* plasmid naming conventions have been described elsewhere (see Table S4 in [19]). In this report we use “plasmid type” to refer to the “PFam32 type” and “plasmid subtype” to refer to organizational variants within such types.

#### Newly discovered plasmid types

Among the plasmids present in the 10 newly analyzed genomes we identify three “new” PFam32 types that are not present in the four previously analyzed genomes of strains B31, JD1, N40 and 297. These plasmids are designated cp32-13, lp28-8 and lp28-9. Additional file 1: Figure S1 shows a RAxML [61] maximum likelihood tree of the known *B. burgdorferi* PFam32 protein types in which the lp28-8, lp28-9 and cp32-13 PFam32 proteins form robust, well-separated branches (the same protein groupings are robustly present in a ClustalX neighbor-joining tree; data not shown). The cp32s present in the previously analyzed complete genome sequences have 11 PFam32 types [19], and the cp32s present in the ten new genome sequences add only one new cp32 type, suggesting that all common *B. burgdorferi* cp32 types are likely now known (if the strains analyzed represent a random sample of extant *B. burgdorferi* diversity). Three genomes, those of CA-11.2A, 72a and 118a, contain the twelfth cp32 type, cp32-13, which was previously known from directed study of *B. burgdorferi* strain CA-15 cp32 partition gene clusters [27] (note that the name “cp32-2” is not used for historical reasons [17, 26]).

**Fig. 2:**
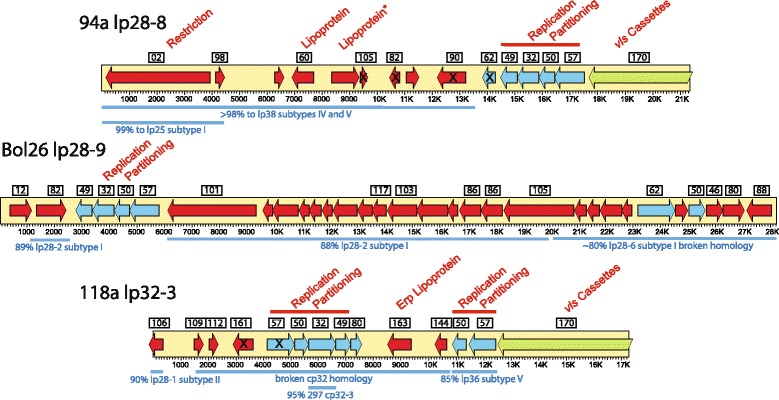
Maps of lp28-8, lp28-9 and lp32-3. The yellow bars represent the plasmids, and the arrows within them indicate most open reading frames in these plasmids; blue arrows are putative plasmid replication/maintenence/compatibility genes, green denotes *vls* cassettes and red are other genes. Black “X”s on arrows indicate pseudogenes. PFams [17, 19] are indicated in the boxes above, and putative function is noted in red text above the map. An asterisk (*) marks the putative lipoprotein gene at ~9 kbp of lp28-8 that is a homolog of *JD1_j07* which encodes a closely related putative lipoprotein; these are ~75% identical to plasmid-encoded proteins PGP088 and BAPKO_6042 of *B. garinii* PBi and *B. afzelii* PKo, respectively; this gene has no homolog in the *B. burgdorferi* type strain B31. The blue bars below note some of the best similarities to other *B. burgdorferi* linear plasmids

Two of the new PFam32 types are encoded by linear plasmids lp28-8 and lp28-9. These are present in isolates 94a and Bol26, respectively (ORF maps are shown in Fig. 2). Their PFam32 proteins are robustly separated from other PFam32 proteins in maximum likelihood and neighbor-joining trees, but the lp28-9 protein is moderately closely related (about 83% identical in amino acid sequence) to its nearest relatives, the lp28-1 type PFam32 proteins (Additional file 1: Figure S1). This lp28-9 relationship is slightly closer than the next most different types, the cp32-8 and cp32-12 PFam32 proteins, which are about 76% identical. The latter two must represent different compatibility types since strains JD1 and 156a carry both cp32-8 and cp32-12 plasmids. These observations prompted the new lp28-9 type name, and although we believe it is likely a novel type, there is no proof at this point that it is truly a different compatibility type from lp28-1 (previously while announcing this sequence in Schutzer et al. [46] we did not make this distinction and originally designated this plasmid as an “lp28-1”).

Although isolate 94a is the only one of the 14 sequenced *B. burgdorferi* genomes that contains an lp28-8 type plasmid, we note that plasmids that encode PFam32’s similar to this lp28-8 protein are present in one of two *B. afzelii* (strain PKo) genomes as well as in the single *B. valaisiana* (strain VS116) and *B. spielmanii* (strain A14S) genomes ([59, 60] and our unpublished analysis). We have reported that the strain PKo lp28-8 harbors a *bor* gene cluster whose products synthesize a peptide that is predicted to be highly posttranslationally modified and is very similar to streptolysin S toxin, and this gene cluster is common the latter three Lyme *Borrelia* species [62], so this plasmid type appears to be more prevalent in these species than in *B. burgdorferi*. The lp28-8 plasmid carries a typical cluster of four plasmid replication/partitioning genes and other genes that are typical of the variable “lp28” group of *B. burgdorferi* linear plasmids; these genes include a PFam01 restriction-modification gene, a PFam60 lipoprotein gene and *vls* cassettes (Fig. 2). An lp28-9 type plasmid is also only present in one of the 14 *B. burgdorferi* genomes analyzed here, but we note that plasmids that encode PFam32 similar to this lp28-9 are present in our sequences of one *B. afzelii* (strain ACA-1) and two *B. garinii* (strains PBr and Far04) genomes ([59, 60] and our unpublished analysis). The gene content of Bol26 lp28-9 is very similar overall to the lp28-2, lp28-6 and lp28-7 group of linear plasmids [19], except that it carries a unique set of partition genes (Fig. 2).

Before this report, each known PFam32 protein type was encoded by either linear or circular plasmids, but not both. However, strains 72a and 118a carry very similar *linear* plasmid sequences that have a cp32-3 type PFam32 gene. These two “lp32-3” plasmids (“lp” for linear plasmid and “32-3” for the cp32-3 PFam32 type). Strain B31 cp32-3 and 118a lp23-3 PFam32 proteins are 96.7% identical (see also Additional file 1: Figure S1), and we note that neither 72a nor 118a carries a cp32-3 plasmid, in agreement with the notion that PFam32 proteins are involved in plasmid compatibility. The two lp32-3 s are syntenic and are extremely similar in nucleotide sequence (98.8% identical over the 11 kbp of non-*vls* cassette DNA). They are not syntenic with any previously known plasmid, but carry genes that encode proteins that are similar to members of various *Borrelia* plasmid-encoded PFams. Figure 2 shows an ORF (open reading frame) map of the strain 118a lp32-3. It carries a set of contiguous *vls* cassettes, several genes whose closest relatives are more typically found on cp32s, including an *erp* family gene (*118a_S17*) [42]. These are arranged in a manner that is not perfectly syntenic with the cp32s. The lp32-3 s also carry regions that are similar to lp28-1 and lp36 of other strains (Fig. 2). Both lp32-3 partition gene clusters contain PFam57, 50, 32 and 49 protein-encoding genes as is the case with most *Borrelia* plasmids, but curiously both of the PFam57 genes in these clusters contain several frameshift mutations; however, there are second, apparently intact, non-contiguous PFam57 genes (*72a_S18* and *118a_S22*) on the lp32-3 plasmids in these two isolates that could perhaps substitute for this frameshifted gene.

**Fig. 3:**
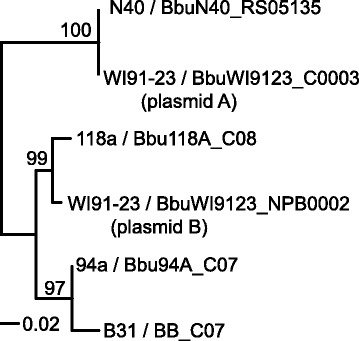
Tree of cp9 PFam57 proteins. A maximum likelihood tree created by RAxML using the PROTGAMMAWAG model [61] of the *B. burgdorferi* cp9 PFam57 proteins is shown with bootstrap values (out of 100 trials) above the lines. A fractional distance bar is shown in the lower left, and strain names followed by the protein locus_tags are shown at the right of each branch. The two strain WI91-23 cp9 plasmids are designated #A and #B (see Additional file 1: Figure S4). A neighbor-joining tree created by ClustalX [96] also places the two WI91-23 in different major branches (not shown)

The compatibility of different *Borrelia* cp9 plasmids has not been studied. The cp9s (and lp5s) contain no PFam32 gene, and so cannot be categorized by their PFam32 type. We note, however, that the genome sequence of isolate WI91-23 appears to contain two cp9s, which must be compatible. All known cp9 plasmids encode PFam57 proteins which are involved in plasmid replication maintenance, partitioning and/or compatibility in other *Borrelia* plasmids [30, 40, 54, 57, 63], and a maximum likelihood tree of these cp9-encoded proteins shows that the two WI91-23 cp9 PFam57 proteins lie on different major branches, but are nonetheless 87% identical (Fig. 3). This difference in sequence could conceivably allow compatibility; however, since the PFam57 protein sequences do not naturally divide into robust, very well separated branches as neatly as the PFam32 proteins, and the specific role of the PFam57 proteins is not known, this idea remains speculative.

Thus, the 29 different PFam32 plasmid compatibility types, in addition to lp5 [17] and the two putative cp9 types described here make it theoretically possible for a single *B. burgdorferi* cell to harbor 32 different *known* plasmid types. Table 1 lists these currently known plasmid types and shows that the presence of these different plasmid types in the 14 sequenced genomes varies greatly. The five most common plasmid types are lp17, lp28-4, lp36, lp54 and cp26; each of these has a representative in all 14 genomes. The least abundant are lp28-7, lp28-8 and lp28-9, each of which is present in only one of the 14 isolates.

**Table 1:** Plasmid frequencies in 14 *B. burgdorferi* isolates

Linear plasmid	Number of isolates^a^	Number of subtypes	Circular plasmid	Number of isolates^a^	Number of subtypes
lp5	2	1	cp9#A	2^b^	1
lp17	14	7	cp9#B	4^b^	2
lp21	3^c^	1	cp26	14	1
lp25	9^a^	4	cp32-1	8^d^	-
lp28-1	7	5	cp32-3	7^d^	-
lp28-2	5	2	cp32-4	11	-
lp28-3	13^a^	5	cp32-5	12^a,^ ^d^	-
lp28-4	14^d^	7	cp32-6	8	-
lp28-5	6	3	cp32-7	10^d^	-
lp28-6	6	4	cp32-8	5^d^	-
lp28-7	1	1	cp32-9	9^d^	-
lp28-8	1	1	cp32-10	10^d^	-
lp28-9	1	1	cp32-11	7	-
lp32-3	2	1	cp32-12	8	-
lp36	14^d^	8	cp32-13	3	-
lp38	11	6			
lp54	14	3			
lp56	4^d^	4			

^a^Number of isolates carrying a plasmid of this PFam32 type among the fourteen completely sequenced genomes. The values shown include the unsequenced lp25, lp28-3 and cp32-5 plasmids present in the original 297, N40 and B31 cultures, respectively (see text)

^b^There are apparently at least two compatibility types of cp9, but it is not known what controls their compatibility (see text)

^c^Includes bulk of lp21 on left end of the chromosome of strain 297

^d^Large (fused) plasmids with two different sets of PFam32 genes are considered as two plasmids in these cases, and each fused portion is included according to its compatibility type

### Organizational variants within individual PFam32 plasmid types

#### Linear plasmid organizational variability

Our previous comparison of the genomes of four *B. burgdorferi* strains, B31, N40, 297 and JD1, showed that some plasmids of the same PFam32 type are very highly conserved among strains, while others can have major differences in genetic content and organization [19]. Even the relatively invariant lp54 exists as three different subtypes whose gene contents vary within the cluster of tandem PFam54 genes near the right end as we have discussed previously (see Additional file 1: Figure S2N here and Wywial et al. [64]). But how many organizational variants of each plasmid type exist in nature? Is the number of variants limited, or are rearrangements occurring sufficiently frequently that nearly every new isolate will have uniquely organized plasmids?

We define organizational “subtypes” within each of the *B. burgdorferi* plasmid types as follows: two plasmids are considered to be of the same subtype if they (i) are syntenic throughout their lengths, (ii) harbor no >400 bp indels (insertions, inversions or deletions) relative to one another (differences in numbers of repeats in short tandem repeat tracts is not considered grounds for separation into different subtypes; see, for example the 63 bp repeat tract lp21 in Additional file 1: Figure S2C), and (iii) have no obvious past inter-plasmid DNA exchanges relative to one another. We name these organizational subtypes with Roman numerals, for example lp17 subtypes I through VII (see below). We note that the lp28-4 and lp36 sequences in strain CA-11.2A appear to be fused end-to-end to form a larger 54.6 kbp linear plasmid (Accession No. CP001480), but they are treated as separate entities for ease of comparison in this report. Additional file 1: Figure S2 presents comparative reading frame diagrams for all 123 linear plasmids from the 14 *B. burgdorferi* genomes. It shows the differences between the subtypes and indicates the organizational subtype of each plasmid. There are 64 different subtypes among these 123 linear plasmids. With the exception of lp5, lp21 and lp32-3 in which little variation has been observed among the few members known, and lp28-7, lp28-8 and lp28-9, each of which is only present once in our strain panel, the linear plasmids in the 14 genomes have between two and eight organizational subtypes. Figure 1 indicates which strains carry which plasmid subtypes, and Table 1 summarizes the number of subtypes of each PFam32 compatibility type are present in our panel of 14 isolates.

**Fig. 4:**
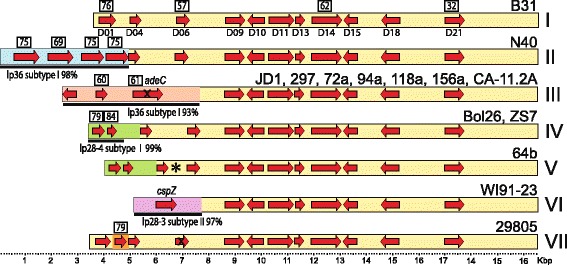
Plasmid lp17 organizational subtypes. Plasmids are represented as in Fig. 2, with different background colors indicating regions of nonhomologous DNA. The organizational subtypes (see text) are indicated by Roman numerals on the right, and isolates that carry each type are indicated above the maps. Some paralogous gene families are indicated in boxes above each map [17, 19], and black bars below indicate some of the similarities to other *B. burgdorferi* linear plasmids

**Fig. 5:**
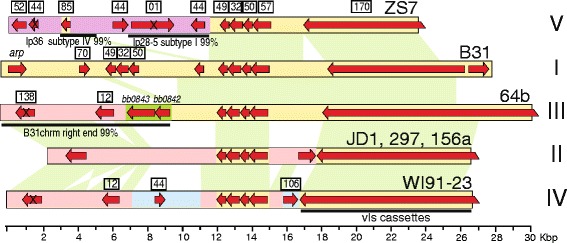
Plasmid lp28-1 organizational subtypes. Plasmids are represented as described in Fig. 4, and green shading between maps connects homologous sequence sections

As examples of the organizational relationships among subtypes, Figs. 4 and 5 show that plasmid lp17, which is present in all 14 genome sequences, has seven different organizational subtypes, and lp28-1, which is present in seven of the 14 genomes, has five structural subtypes. Curiously, all the lp17 organizational differences are all present as different sequences at the plasmid left ends. These alternate left-end sequences are not novel, but are instead similar to sequences present in other *B. burgdorferi* plasmids. As we previously reported, the N40 and JD1 lp17s (subtypes II and III, respectively ([17, 19] and the analysis in this report, [29, 42]) harbor alternate left end sequences that are very similar to two different parts of lp36 (which we now know is lp36 subtype I; Additional file 1: Figure S2L). For example, bps 300–1500 of the type II N40 lp17 are 99.3% identical to bps 33169–34379 of the type I B31 lp36, and bps 1574–2207 of the type III JD1 lp17 are 93.4% identical to bps 10021–12222 of B31 lp36. We show here that in addition, lp17 subtypes IV and VI have left end sequences that are very similar to terminal or near-terminal sequences of plasmids lp28-4 subtype I and lp28-3 subtype II, respectively; for example, the type IV ZS7 lp17 bps 42–1244 are 99.5% identical to B31 type I lp28-4 bps 1–1203, and, ignoring three deletions in the 94a plasmid relative to the WI91-23 plasmid, the type VI WI91-23 lp17 bps 33–2667 are 98.1% identical to the type II 94a lp28-3 bps 1–3995). We also note that lp17 subtype VII has a divergent PFam79 gene inserted near its left end (Fig. 4). Similarly, the five types of lp28-1 plasmids all carry *vls* cassettes and closely related partition gene clusters, but also have very substantial regions of difference that are often very similar to parts of other linear plasmids (Fig. 5).

Have all the linear plasmid subtypes been identified? Nearly identical plasmids of the same subtype in different isolates are clearly not uncommon, as about half of the linear plasmids belong to subtypes that have more than one member (Additional file 1: Figure S2). On the other hand many subtypes are currently represented by a single plasmid (*e.g*. lp28-1 subtypes I, III, IV and V; Fig. 5). The latter observation suggests that subtype delineation might currently be far from complete, and a large number of additional subtypes might exist. However, a substantially larger fraction of extant plasmid types *might* be known than such a calculation based on random plasmid distribution would suggest, because the 14 strains whose genome sequences we determined were picked to be as diverse as possible and to represent a significant fraction of the spectrum of multilocus sequence (MLST), rRNA and OspC diversity of *B. burgdorferi* (above). If, for example, plasmid contents are fairly constant *within* each of the different rRNA/OspC lineages, then a substantial fraction of the extant plasmid subtypes might be known (see below). On the other hand, some plasmid types and subtypes may be common only in particular geographical regions or in ospC types that have not yet been sampled by sequence determination. Analysis of more varied isolates will be required to answer these questions.

#### *Linear plasmid gene content in* B. burgdorferi *isolates*

There is substantial variation in the plasmid content of different *B. burgdorferi* isolates (Fig. 1). The fact that many plasmid genes belong to paralogous families means that most isolates carry members of nearly all of the *B. Burgdorferi* gene families (see the detailed analysis of strains B31, JD1, 297 and N40 in Casjens et al. [19]). However, the rearrangements that have given rise to the different plasmid subtypes have often placed the most highly related gene pairs on different plasmid types in different isolates. Additional file 1: Table S1 lists the locations of some of the important and better-studied linear plasmid gene types in each of the 14 strains. A majority of these genes are found on several different plasmid types. However, in spite of the past duplicative rearrangements that have happened to these plasmids, some genes are typically only present in one copy and always on the same plasmid type, for example *pncA* (nicotinamidase [65]) on lp25*,* and *adeC* (adenine deaminase [25]) and *fbp* (fibronectin binding protein [66]) on lp36. Interestingly, the *adeC* (PFam61) gene is present and intact on all lp36 plasmids, but on plasmid lp17 subtype III (Fig. 2 and Additional file 1: Figure S2B), defective *adeC* paralogues have suffered several reading frame disrupting mutations in all seven isolates that carry the latter plasmid. The *cspZ* gene, which encodes a complement regulation protein [51], is typically present in one copy on lp28-3, but in strain WI91-23 a second apparently intact copy is on lp17. The cyclic di-GMP binding protein gene [67] is present on two different plasmids, lp28-1 subtype V and lp28-5 subtype I, in different isolates, and *arp* (arthritis-related protein [68]) is found on four different plasmid types, lp28-1 subtype I, lp28-4 subtype III, lp28-5 subtype I and lp36 subtype II (Additional file 1: Figures S2E, H, I and K). Thirteen of the 14 strains carry a set of *vls* cassettes; this region is absent only from CA-11.2A. The location of the *vls* cassettes is quite variable, and they lie on five different linear plasmid types that include 10 different subtypes: lp28-1 (all five subtypes carry *vls*), lp28-3 (one subtype), lp36 (two subtypes), lp28-8 (one subtype) and lp32-3 (one subtype) (Fig. 2 and Additional file 1: Figure S2E, G, L and K). We note that no *vlsE* gene, which encodes a hyper-variable outer surface protein and is modifed by *vls* cassette sequences (and presumably their expression locus, *vlsE*, which encodes a hyper-variable outer surface protein [69]), was found in the sequencing libraries of any of the 14 strains, most likely because a region between the *vls* cassettes and the *vlsE* gene region appears to be unclonable in plasmid DNA libraries [70, 71]. Restriction-modification genes are rather plentiful in all but one (Bol26) of the 14 isolates, and 156a and 118a each have four *apparently* intact plasmid-borne genes that encode such proteins. It is difficult to assess the relevance of the absence of *vls* cassettes in CA-11.2A, of *cspZ* in 72a, 29805 and Bol26, or of *arp* in 64b, Bol26, WI91-23, 297 and ZS7 due to the ease with which linear plasmids are lost in culture. Answers to these questions may have to wait until all plasmid subtypes can be unambiguously delineated for *B. burgdorferi* bacteria in wild-caught ticks, *i. e.*, conditions in which the bacteria have not been propagated in culture.

#### Circular plasmid organizational variability

There are three general kinds of circular plasmids present in the 14 *B. burgdorferi* complete genomes, the cp9, cp26 and cp32 families of plasmids. Since only these kinds are found in the 14 genomes, additional kinds of circular plasmids, if they exist, must be uncommon. The circular cp26 plasmids are organizationally identical in all 14 sequenced genomes, and we do not define organizational subtypes for the cp32s. Although the latter are syntenic overall, they do have significant gene content differences in their four variable regions [19] and they exhibit a few large indels (below); however, homologous recombination appears to have shuffled the combinations of these regions to the point that a majority of the individual cp32 plasmids would define a unique “subtype” [19, 42]. For example, each of the 32 cp32s in strains B31, JD1, N40 and 297 has a different combination of variable region “alleles” [19]. Thirteen of the 89 fully assembled cp32 sequences contain substantial (8 to 16 kbp) deletions, and one has a 5.6 kbp inversion; these are delineated in Additional file 1: Figure S3 and indicated in Fig. 1). None of these rearrangements removes any of the partitioning genes or any of the studied “lysogenic conversion” genes that are expressed from these prophage plasmids (*rev, mlp, bapA,* and *erp* gene groups) [29, 42, 72]. The 297 and 156a cp32-7 s have identical deletions, as do the 118a and 72a cp32-5 s; each of the other nine deletions is unique. Since these deletions were likely created by nonhomologous recombination events [17, 19], each of these pairs of identical deletions is almost certainly descended from a single ancestral deletion event (see below).

In addition, two types of cp32 rearrangements are found that are not simple deletions or inversions. As previously reported, in strain B31 a cp32-10 is integrated into an lp56 plasmid by nonhomologous recombination [17]. The linear lp56 present in strain 94a has no such insertion and is 99.5% identical over about 95% of its length to the non-cp32 portion of the B31 lp56 (the two plasmids only differ by a few kbp of sequence at their right ends, far from the cp32 insertion site; Additional file 1: Figure S2O). Thus, the 94a lp56 almost certainly represents the parental type of plasmid lp56 into which the cp32 integrated in strain B31. The second type of cp32 rearrangement is fusion between full-length cp32s of different compatibility types; such dimer plasmids are present in strains JD1, ZS7, 64b and 118a (noted in Fig. 1 and Table 1) and their formation appears to have been mediated by homologous recombination, since no novel sequence joints are present. Thus, of the cp32 sequences, 14 have been modified by recent nonhomologous rearrangements, and 78 are apparently intact (the latter include the seven full-length cp32 sequences that are present in heterodimer circles). Finally, we note that, since the strain 118a and 72a lp32-3 sequences are syntenic and about 99% identical in sequence over most of their length (above and Additional file 1: Figure S2K), the ancestral exchange between a cp32-3 and a linear plasmid(s) to generate lp32-3 most likely happened only once in our strain panel (also see below). Compared to most of the linear plasmids, relatively few *nonhomologous* rearrangement events have been involved in generation of the extant cp32 organizational diversity.

The circular plasmid type cp9 has three organizational subtypes that are shown in Additional file 1: Figure S4. Subtypes II and III have about 300 bp of apparently non-protein-coding DNA that replaces the B31 PFam63 *revB* gene (*B31_c10* [73]) and subtype III’s plasmid maintenence/partition gene cluster (encoding PFam57, 50 and 49 proteins) is inverted relative the other two subtypes. All three subtypes have apparently intact genes that encode the exported EppA protein (*B31_c06* [74]).

### Rare genomic rearrangements (mosaic boundaries) can track *Borrelia* plasmids

#### The nature and utility of rare genomic rearrangements

As discussed above, *B. burgdorferi* plasmid subtypes are mosaically related to one another in that they have patchy or mosaic sequence relationships in which two plasmids can have patches of very high sequence similarity (often several kbp that are nearly identical) that are adjacent to very different (often nonhomologous) sequences. These highly related patches are bounded by “novel sequence joints” or “mosaic boundaries.” Such mosaic boundaries have been created by rare genomic rearrangements that are apparently mediated by nonhomologous recombination events. We previously discussed the sequences at some specific mosaic boundaries in the B31, N40, 297 and JD1 plasmids [17–19], and the numerous new mosaic boundaries discovered here in the plasmids and in the chromosomal right-end extensions of ten new genomes have similar characteristics; *i.e.,* when it can be determined they appear to have been generated by nonhomologous recombination (analysis not shown).

We have argued that these linear plasmid rearrangements appear to occur randomly since the novel sequence joints that result are often present within genes, and it seems very unlikely that there is a positive selection for the resulting broken genes [17–19]. There are 3 × (number of bp)^2^ possible different random break and rejoin points or mosaic boundaries that can form when any sequence is rearranged with an identical sequence (see Additional file 1: Figure S5). Therefore, since there are on average about 250,000 bp of linear plasmids in *B. burgdorferi*, there are about 3 × 250,000^2^ bp = 0.19 trillion possible different random novel joints can in theory be formed by DNA exchanges within the linear plasmids. It seems prohibitively improbable that identical novel sequence joints will be found that were created independently, and we believe that identical novel joints found in more than one isolate are descendants of a single ancestral rearrangement event. By a similar argument, it is also extremely improbable that a random nonhomologous event would be precisely reversed by another such event. In practical terms the extant novel joints, unlike simple base pair changes, very likely happened only once and do not revert precisely (see Gupta [75] for a more general discussion).

The rate of these rearrangements relative to the rate of nucleotide substitutions can be estimated. Using lp17 as an example, we find 17 SNPs in a multiple alignment of 738 nucleotides of PFam32 genes from the *B. burgdorferi* genomes. All SNPs are synonymous except one Ile to Leu nonsynonymous difference. Assuming that all synonymous SNPs are selectively neutral, the rate of synonymous nucleotide substitution (commonly known as *K*_*S*_) is estimated to be 0.0994 substitutions per synonymous site by using the program PAML [76]. Using a combination of five genes on lp17 (BB_D09, BB_D13, BB_D14, BB_D18, and BB_D21) yielded a similar synonymous (neutral) substitution rate of 0.107 per site. Among the 14 lp17 plasmids we identify 11 novel sequence joints within their average 17000 bp length (ignoring small indels <80 bp and variations in the length of the short repeat tract at about bp 13200 in strain B31 lp17, and assuming that indels were formed by a single out-of-register nonhomologous recombination event). These lp17 novel joints are indicated in Additional file 1: Figure S2B. Therefore the rate of rearrangements among these genomes is approximately 11/17000 = 0.00065 mosaic boundaries per base pair on lp17. We thus estimate that nonhomologous rearrangement of lp17, and presumably the other linear plasmids, in *B. burgdorferi* is substantially less frequent than nucleotide substitution, occurring at a minimum rate of approximately 0.65% (0.00065/0.0994) of the rate of neutral nucleotide substitution. This corresponds to a minimum value for the ratio of their rates of formation, since novel joints seem more likely to be selected against (and therefore not be present in extant sequences) than simple bp changes; however, our observation that novel joints are often present within genes and should inactivate those genes suggests that they can often be tolerated even when genes are broken during their formation [17–19]. We also note that homologous recombination between regions of similar sequence among the plasmids could in theory create new *combinations* of these novel joints at a much faster rate than their rate of formation. The linear plasmid novel sequence joints should serve as excellent genetic markers in the study of *B. burgdorferi* population structure, possible plasmid exchange and plasmid evolution.

#### Exchange of sequences between linear plasmids and the chromosome

**Fig. 6:**
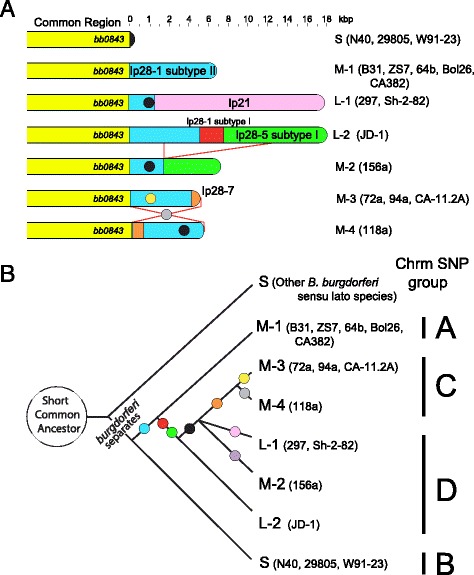
*B. Burgdorferi* chromosomal right end extensions. **a** Aligned chromosomal right end maps drawn to scale. The different colors represent very high similarity to the indicated plasmids. The black and yellow circles indicate 263 bp and 324 bp overlapping deletions, respectively, relative to the B31 chromosome. The right-end extension names and strains that carry them (in parentheses) are indicated at the right. The thin red lines with a gray circle between the L-2 and M-2 and M-3 and M-4 chromosomes indicate a large putative deletion and inversion, respectively. **b**. Evolutionary tree compatible with terminal replacements and rearrangements. Major rearrangements, assumed to have occurred on the chromosome and not on plasmids before they recombined with the chromosome (see text), are denoted by the small colored circles: blue, lp28-1 subtype II addition; red, lp28-1 subtype I addition; green, lp28-5 subtype I addition; black, 263 bp deletion in lp28-1 type II sequence; orange, lp28-7 addition; gray, 7 kbp inversion; purple, 12 kbp deletion; pink, addition of lp21 sequences; and yellow, enlargement of 263 bp deletion (above) to 324 bp. The right end extension names and strains that carry them (in parentheses) are indicated at the tips of the branches, and the chromosomal SNP types (see text) are given at the right. Strains 297, Sh-2-82 and CA382 were not included in the SNP analysis but fit in the SNP groups according to the facts that, like B31 in SNP group A, CA382 has a type A ospC (A. Barbour, personal communication); 297 and Sh-2-82 are both ospC type K, and 297 has lp54 and cp26 plasmids that are most closely related to the SNP group D strains (see text) [48]

Some but not all *B. burgdorferi* chromosomes have linear plasmid-like sequences between 7 and 20 kbp long covalently attached to their right ends [77]. Previously three different terminal attachments of this kind were known, those typified by isolates B31, JD1 and 297 [19]. Among the 15 chromosomes whose sequences are now known, including the 10 isolates whose plasmids are newly described here and the chromosome of strain CA382 (A. Barbour and R. Lane, Accession No. CP005925), twelve have right-end extensions that increase the total to six different right-end sequence extensions (Fig. 6a). All six extension types are composed of sequences that are closely related to linear plasmid sequences. For example, the blue lp28-1-like sequences in Fig. 6a, although not all of the same length because of partial replacements in the different strains, are all >99% identical to each other where homologous sequences are present, and the overlapping green lp28-5-like sequences in strains JD-1 and 156a are (ignoring three indels) >99% identical. Similarly, the relationships between the chromosome extensions and their “parent” plasmids are very close. For example, the strain B31 right-end chromosomal extension (blue in Fig. 6a) and the strain 297 lp28-1 homologous regions are >98% identical, the strain 297 extension (pink in Fig. 6a) and B31 lp21 homologous regions are >99% identical (ignoring differing numbers of repeats in the 63 bp repeat region), and the strain JD1 extension (green portion in Fig. 6a) and the N40 lp28-5 homologous regions of are >99% identical. The right ends of strains 156a, 118a and CA-11.2A typify three new types; 94a and 72a right ends are the same as CA-11.2A, and 118a is similar to CA-11.2A, but its terminal sequence has suffered an ~7 kbp inversion. If assumptions are made that (i) the ancestral state had no plasmid-like sequences joined to the chromosome (likely correct since the several closely related *Borrelia* species where this is known have no such right end extensions ([4] and our unpublished analysis), and (ii) the novel sequence joints between the plasmid-like and resident chromosomal sequences were created when the plasmids recombined (apparently nonhomologously) with the chromosome, several possible sequences of major events can be deduced for the creation of these extensions. Figure 6b depicts one possible order for these events. Clearly, even if this particular scenario is not entirely correct, there must have been multiple sequential plasmid sequence addition events on several of the chromosomes. Our previous maximum-likelihood tree analysis of chromosomal SNPs (single nucleotide polymorphisms) identified four chromosomal subgroups that are clustered with very strong support - group A (strains B31/64b/ZS7/Bol26), group B (N40/29805/WI91-23), group C (72a/94a/118a/CA-11.2A) and group D (JD1/156a) [48]. SNP groups A, B, C and D correspond to the right end types M-1, S, M3 + M-4 and L-1 + L-2 + M-2, respectively (Fig. 6b). No right end organizational type is found in more than one SNP group, and the branching order of the right end tree in Fig. 6b is compatible with the chromosomal SNP tree. Strains 297, Sh-2-82 and CA382 were not included in the chromosome SNP analysis, but their chromosomal right ends have been analyzed ([77] and Accession No. CP005925), and they fit appropriately in Fig. 6b if their OspC type is considered to be indicative of their chromosomal SNP type (see below). The concordance of the chromosome SNP groups and chromosome right end types suggests that the different right end extensions have not been horizontally transferred between chromosomal constant region lineages.

We also note that the sequence of plasmid lp28-1 subtype III in isolate 64b contains closely related homologs of a putative arginine catabolism gene (*b31_0842,* truncated) and a transporter gene (*b31_0843*) (green shading in Additional file 1: Figure S2E), which are the two rightmost genes in the “constant region” of the chromosome in all *B. burgdorferi* strains that have been examined. In addition to this normally chromosomal sequence, the adjacent ~7 kbp at the left end of this plasmid are 98.9% identical to the contiguous plasmid-like sequences at the right end of the strain B31 chromosome (Additional file 1: Figure S6). This is parsimoniously explained if the leftmost ~9.5 kbp of lp28-1 subtype III is actually derived from the right end of an M-1 type chromosome. Thus, genetic information has apparently been transferred from the right end of the chromosome onto a linear plasmid.

#### Chromosomal lineage correlates with plasmid subtypes

Are linear plasmid subtypes limited to particular chromosomal lineages, or are they horizontally exchanged relative to the chromosome at an appreciable rate? In spite of numerous examples of past horizontal exchange of various smaller regions [19, 27, 42, 64, 78–87], it is not known if whole plasmids exchange between *B. burgdorferi* lineages at a significant rate. Previous analyses have shown a strong correspondence between the chromosomal SNP pattern and the SNP pattern of plasmid cp26 (and its encoded OspC protein) as well as with the SNP pattern of plasmid lp54 [48, 78, 79, 88]. This indicates that reassortment of the chromosome and these two plasmids is not common [48, 78, 79, 88]. But failure of cp26 or lp54 to exchange does not mean that other plasmids may not exchange more rapidly. By considering the distribution of the *B. burgdorferi* linear plasmid subtypes (each of which has unique mosaic boundaries as discussed above), we can begin to examine the natural population dynamics of the linear plasmids of *B. burgdorferi*.

*B. burgdorferi* chromosomal variation studies have noted a number of subgroups within this species [48, 64, 83, 88–90], and in particular our recent analysis of the sequenced chromosomes showed that strains 297/156a, 72a/118a and ZS7/Bol26 represent three pairs of particularly closely related chromosomes [48]. The chromosome of 297 has not been completely sequenced, but it is rather closely related to 156a by its similarity to 156a rRNA IGS1 sequence, multilocus sequence typing analyses, right end structure (above and Fig. 6), as well as SNP analyses of the two “non-exchanging” (above) plasmids cp26 and lp54 [48, 84, 87].

**Fig. 7:**
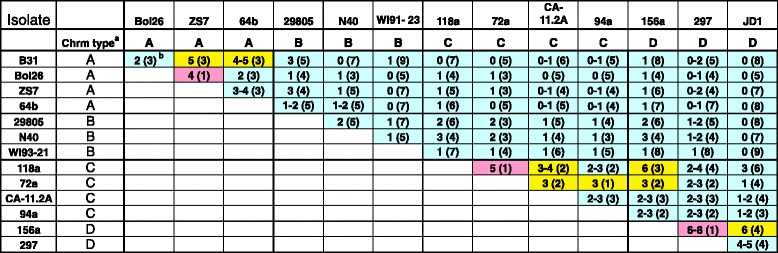
Comparison of linear plasmid content of *B. burgdorferi* isolates. Values outside parentheses are the number of plasmid subtypes in common in the pairwise comparisons (uncertainty in some cells is the result of uncertainty of the subtype of a few linear plasmids due to incomplete terminal sequences - see legend to Fig. 1). Values in parentheses are the number of plasmids of the same PFam32 compatibility type that are different subtypes in the pairwise comparison (assuming plasmids of uncertain subtype are not the same in the strain pair being compared). Thick lines separate isolates with different chromosomal SNP types (see Fig. 6 and text). Cell color indicates relatedness of linear plasmid contents as follows: pink, number of plasmids of same subtype/number of plasmids of different subtype is ≥4; yellow, same as pink only values ≥1 and <4; blue, same a pink only values <1

The currently available sample size is small, but even at this early stage it is clear that many linear plasmid subtypes are not randomly distributed among the chromosomal types. For example lp17 and lp36 plasmid subtypes with multiple members are distributed as follows: In lp17 both subtype IV plasmids are present in chromosomal SNP group A strains, and the seven subtype III plasmids are limited to two of the four groups, C and D. Similarly, all four lp36 subtype I plasmids are found in group A, all three subtype II plasmids are in group D, and the two subtype III plasmids are found in group C. In addition, the cp32-2 deletions A and I (Fig. 1 and Additional file 1: Figure S4) are limited to chromosomal groups D and C, respectively. Figure 7 shows the number of cognate and noncognate linear plasmid subtypes present in all pairwise comparisons of the 14 *B. burgdorferi* genomes (ignoring plasmids whose PFam32 type is not present both members of the pair). These comparison values were classified into three relatedness classes; highly related, moderately related and less related (pink, yellow and blue cells, respectively, as defined in the legend of Fig. 7). Isolates 297 and 156a have the most similar linear plasmid contents, with 6 to 8 plasmid types with identical subtype in both strains and only one plasmid type with different subtypes in the two strains. (The uncertainty derives from the fact that the subtypes of 297 plasmids lp28-3 and lp28-4 cannot be determined precisely due to unsequenced regions near some plasmid ends; nonetheless, their sequenced regions are the nearly identical to the parallel 156a plasmids; Additional file 1: Figures S2G and H). The isolates with the least related plasmid contents are JD1 and WI91-23, where no identical plasmid subtypes among the nine different types that are present in both strains. Strikingly, the three pairs of strains whose plasmid subtypes are most similar, 297/156a, 72a/118a and ZS7/Bol26 (the only pink cells in Fig. 7), are the same three pairs identified as closest chromosomal relatives (above). These three pairs have 6–8, 5 and 4 identical plasmid subtypes, respectively, and all three have only one case of different subtypes of the same plasmid type (lp28-3 subtypes I and V in ZS7 and Bol26; lp36 subtypes II and IV in 72a and 118a, and lp28-5 subtypes I and III in 156a and 297, respectively). Of the eight moderately related strain pairs (yellow cells in Fig. 7), six are pairs from within the same chromosomal group (*e.g*. the chromosomal group D strains JD1 and 156a have 6 linear plasmid subtypes in common and 4 that are different, and the chromosomal group A strains B31 and ZS7 have 5 subtypes in common and 3 that are different). The only two moderately related inter-group pairs are 156a/118a and 156a/72a are both comparisons between group D and C strains, and we note that the A/B chromosomes and C/D chromosomes form two more closely related groups [48]. Of the 73 inter-group chromosomal comparisons in Fig. 7, 71 are in the least related class (blue cells). No close linear plasmid subtype relationships among the three strains in chromosomal group B were found, suggesting that the plasmids in the three strains in this group are more diverse than the strains within the other three chromosomal lineages. We also note that *all* pairwise comparisons show at least one pair of plasmids of different subtype from within the same PFam32 type.

In spite of the above correlation between chromosome type and plasmid content, results from this small sample do not rule out the possibility that horizontal transfer of whole linear plasmids has occurred among the lineages represented by the 14 genomes; for example, lp28-4 subtype II is present along with at least one other lp28-4 subtype in each of the four chromosomal SNP groups; however, each of the other six subtypes is present in only one chromosomal SNP group and so could have arisen *within* that group’s lineage. Indeed, the distribution of each plasmid and its subtypes among the 14 isolates can be explained by a scheme that includes only linear descent and creation of new subtypes *within* the different chromosomal groups, where all subtypes (*i. e*., mosaic boundaries) are created only once. The current analysis does not identify any unambiguous example of whole linear plasmid transfer, and we conclude that, although it might occur occasionally, the rate of such transfer is not high enough to mask the observed parallels between chromosomal type and plasmid subtype content. This interpretation, while admittedly still somewhat speculative, in turn suggests that generation of novel plasmid subtypes has occurred multiple times within each of the four chromosomal SNP lineages since the evolutionary separation of these lineages.

Any correlation between geographic location or animal host and plasmid content remains to be clarified. Our study includes individual Wisconsin, California, Germany and Italy isolates, in addition to ten southern New England/New York isolates. A number of the identified plasmid subtypes are unique to isolates of a particular geographic region; for example, nine of the Wisconsin isolate WI91-23’s 11 linear plasmid subtypes are unique to this isolate, three of the California isolate CA-11.2A’s six linear plasmid subtypes are unique, and lp17 subtype IV, lp28-1 subtype V, lp28-3 subtype V and lp28-9 are present only in the European isolates. However, there are also numerous unique plasmid subtypes in individual New England isolates (*e.g*. lp17 subtypes II and VII), and on the other hand, there are also identical lp17 subtypes in geographically distant locations (*e.g.* lp17 subtype III in New England and California isolates; and lp36 subtype I in New England and European isolates). Clearly, analysis of plasmid subtypes in many more isolates from different geographical regions and from various hosts will be required to understand the population biology of *B. burgdorferi* in terms of the relationship of plasmid content to geographic location, animal host, *etc.*

#### *Plasmids in* B. burgdorferi *isolates with closely related ospC genes*

The four chromosomal SNP types discussed above represent very broad lineages. Do narrower lineages exhibit perhaps even stronger linear plasmid uniformity? Each chromosomal SNP type includes isolates that have different rRNA IGS1 spacer sequences (intergenic rRNA region on the chromosome) and different OspC types (*ospC* gene on the cp26 plasmid), both of which have been used to classify *B. burgdorferi* into narrower groups [78, 79, 88]. Most of the *B. burgdorferi* genome sequences are from strains that are different IGS1 and OspC types, so it is therefore perhaps not surprising that all of these 13 isolates have different plasmid contents. But might isolates with identical rRNA IGS1 sequences or identical OspC types have more similar plasmid contents? Information is rather meager at present, but we have identified 27 strains where IGS1 sequences and OspC types are known and for which there is some information available on linear plasmid content. In addition to the 14 complete genomes, IGS1 and *ospC* sequences have been determined for strains EMC-NY-86, Sh-2-82, 2591, 19535, 19678, 26815, 26816, 27985, 28534, 29592, 29850, 29968 and 30757; see Methods). Right end chromosome extension lengths were also determined for these isolates by Southern analysis as described in Casjens et al. [91] (data not shown). Table 2 shows that these 27 isolates fall into eight chromosomal rRNA IGS1 types and eight parallel OspC types; this correlation between IGS1 type and OspC type has been noted previously [6, 92]. Table 2 shows that each IGS1/OspC group has an apparently invariant chromosomal right end extension, suggesting that horizontal transfer or invention of new right end extensions *within* these groups is not a common event (also see above). Linear plasmid sizes for lp36, lp38 and lp56 related plasmids are also shown in Table 2). These plasmids were chosen here because they are the most variable in length; plasmid size differences of several kbp are ignored so any correlations would not be confused by measurement inaccuracies. There is no evidence in Table 2 of size differences in these linear plasmids within any of the eight groups. Thus, although this information is certainly incomplete at this time, these findings are consistent with the idea that these three linear plasmids are uniform within a single IGS1/OspC type. In addition, Stevenson and Miller [27] found that strains B31 and BL206 (both rRNA IGS1 lineage 1, OspC type A; [93]) have very similar cp32 contents by analysis of the sequence of the variable regions of their cp32 plasmids, as do strains 297 and Sh-2-82 (both IGS1 lineage 2, OspC type K). Additional study is clearly necessary, but this preliminary information suggests that isolates with very similar IGS1/ospC genotypes may well usually have very similar plasmid contents.

**Table 2:** Comparison of *B. burgdorferi* isolates with identical IGS1 sequences

Chromosome right							Strain reference
Strain	IGS1^a^	OspC^a^	end^a^	lp38^b^	lp36^b^	lp56 ^b^	
B31^c^	1	A	M-1	38(I)	36(I)	56(I)	[101]
30757	1	A	M	–	36	56	[102]
26816	1	A	M	–	–	–	[102]
27985	1	A	M	38	36	56	[102]
19535	1	A	M	–	36	–	[103]
132b	1	A	M	–	–	–	[83]
297^c^	2	K	L-1	27(IV)	24(II)	np	[104]
28534	2	K	L-1	–	24	–	[102]
29968	2	K	L-1	–	–	–	[102]
Sh-2–82	2	K	L-1	–	–	–	[105]
136b	2	K	L	–	–	–	[83]
163b	2	K	L	–	–	–	[83]
64ab^c^	3	Ba	M-1	38(I)	36(I)	np	[83]
160b	3	Ba	M	–	–	–	[83]
EMC-NY-86	3	Bb^d^	M	38	–	–	[103]
19678	3	Bb^d^	M	38	35–36	–	[103]
29850	4	N	S	–	–	–	[102]
26815	4	N	S	–	–	–	[102]
CA-11.2A^c^	19	Db	M-3	28(V)	26(VII)^e^	29(III)	[106]
121a	19	Db^d^	M	–	–	–	[83]
29805^c^	6	M	S	38(I)	36(V)^f^	np	[102]
2591	6	M	S	–	–	–	[102]
WI91-23^c^	7	Ia	S	38(III)	30(I)	28(IV)	[107]
HB19	7	Ia	S	38	30	–	[104]
97b	7	Ia	S	–	–	–	[83]
N40^c^	9	E	S	37(II)	30(IV)	np	[108]
29592	9	E	S	37	30	–	[102]

^a^IGS1 and OspC sequences are defined as in Travinsky et al. [88] and Barbour and Travinsky [79], respectively. Chromosome right ends are named as in Fig. 6b, except right end type names M and L without a number (*e.g.*, M *vs.* M-1) indicate that right end restriction fragments have the same size as other group members by Southern analysis, but the termini have not been sequenced. In the case of strains 28534 and 29968 additional restriction mapping confirmed that these strains have L-1 type chromosome right ends, although they have not been sequenced

^b^In the table, (i) numbers in the lp38, lp36 and lp56 columns indicate the size of the observed electrophoretic plasmid DNA band in kbp, and in the sequenced genomes the plasmid subtype is given in parentheses ([8] and data not shown); (ii) dashes (−) denote that the plasmid is either missing or did not react with the strain B31 templated Southern probes that were used ([8] and data not shown); and (iii) “np” indicates that a plasmid of this type is not present in the genome sequence.

^c^Complete genome sequence is known

^d^Sequence is closest to this OspC type, but is not identical to the prototypical member of that type

^e^26 kbp is the lp36 portion of the apparently end-to-end fusion of lp36 and lp28-4 plasmids in strain CA-11.2A (see text)

^f^Strain 29805 lp36 is similar in size to that in B31 but has quite different sequence organization (see Additional file 1: Figure S2L and text)

## Conclusions

We have analyzed the sequences of the 236 plasmids present in the genomes of 14 *B. burgdorferi* isolates. Since 21 plasmid types were found in the first genome sequenced [17, 45], seven more in the next three genomes [19], and only four more in the final ten genomes analyzed, it appears that the probability of finding new types is diminishing rather rapidly with the analysis of additional isolates. Here we identified four new plasmid putative compatibility types (lp28-8, lp28-9, cp32-13 and a new type of cp9) in the ten newly analyzed genome sequences, which brings the total to 32 known types. However, since relatively few strains from outside New England have been analyzed in this detail, the possibility remains that more novel plasmid types will be found in other geographical regions.

We showed here that most of the linear plasmids are present in the *B. burgdorferi* population as multiple organizational subtypes, and the number of these subtypes is projected to be fairly large but limited. The mosaic sequence boundaries in these plasmids represent rare and essentially non-revertable genetic markers that can be used to evolutionarily and epidemiologically track plasmids. Previous studies using small numbers of genetic markers have noticed anecdotal correlations between plasmid content and chromosomal genotype [6, 92], and the more comprehensive findings presented here support this notion. Among the 14 sequenced genomes and 13 additional isolates that we examined by other means, isolates with more closely related chromosomes and cp26 plasmids have more closely related linear plasmid contents. These findings indicate that, although horizontal transfer of linear plasmid genetic material between *B. burgdorferi* lineages clearly does occur in nature (e. g. [64, 87]), there is as yet no evidence of whole linear plasmid transfer in the wild. We note that a number of the plasmids appear to be prophages or related to prophages [17, 28] and so might be expected to transfer as whole entities. In particular, the cp32 circular plasmids have been shown to be transferred, apparently as phage virions, between strains in the laboratory [94]. However, the cp32 plasmids have apparently undergone many apparently homologous exchanges that would tend to obscure past whole plasmid transfer [17–19]. In any case, plasmid transfer is not frequent enough to disrupt the correlation that exists at this stage of analysis between chromosomal IGS and cp26 OspC markers and linear plasmid organizational subtypes. A complete picture of *B. burgdorferi* population structure will certainly require an understanding of the complete gamut of plasmid organizational subtypes and their distribution in all chromosomal lineages.

## Methods

We have previously described the sources of the isolates whose genomes have been sequenced and the methods used for sequence determination [46, 48]. Nucleotide and protein sequence alignments were created by BLASTp and BLASTn [95], ClustalX [96] and DNA Strider [97], and matrix comparison plots were created by DNA Strider [97] and Gepard [98]. Neighbor-joining trees were created by ClustalX [96], maximum likelihood trees were created by RAxML [61], and trees were drawn by NJPlot (http://doua.prabi.fr/software/*njplot*.html) and FastTree [99]. For analysis of sequence substitution rates of genes on the lp17 plasmid, we calculated dN and dS rates using PAML with the uniform dN/dS ratio model (NSsites = 0) [76]. In all cases computer programs were used with default settings unless otherwise indicated.

Plasmid subtypes were determined and analyzed as follows: After determination of the encoded PFam32 protein type using BLASTp sequence similarity and ClustalX tree analysis (see Additional file 1: Figure S1) BLASTn sequence comparisons and DNA Strider (usually initially at a window stringency setting of 15 out of 23 bp identities) and Gephard dot plot plasmid comparisons were used to identify groups of very similar plasmids (subtypes) that do not have large indels relative to one another. Long regions of similarity between different plasmid types and subtypes were typically identified first by dot plot analysis and then characterized in more detail by nucleotide sequence alignment of specific regions using BLASTn and DNA Strider.

Some *OspC* gene and rRNA IGS1 sequences were determined by dideoxynucleotide sequencing methodology after PCR amplification using the universal primers described by Qiu et al. [83] and Bunikis et al. [100], respectively. The resulting amplified DNA was sequenced by dideoxy-sequencing methods at the University of Utah Core Sequencing Facility (data not shown).

## Additional file

Additional file 1: Figure S1.
*B. burgdorferi* plasmid PFam32 protein tree. **Figure S2.** Comparative maps of linear plasmids in 14 *B. burgdorferi* isolates. Maps of the following linear plasmids are shown in the following panels: A, lp5; B, lp17; C, lp21; D, lp25; E, lp28-1; F, lp28-2, lp28-6, lp28-7 and lp28-9; G, lp28-3; H, lp28-4; I, lp28-5; J, lp28-6; K, lp32-3; L, lp36; M, lp38; N, lp54; O, lp56. **Figure S3.** Deletions in cp32 circular plasmids. **Figure S4.** Comparative cp9 plasmid maps. **Figure S5.** All possible recombination products. **Figure S6.** Ancestral transfer of genetic material from the *B. burgdorferi* chromosome right end to a linear plasmid. **Table S1.** Linear plasmid locations of selected genes. (PDF 3869 kb)

## Abbreviations

ORF: open reading frame
PCR: polymerase chain reaction
SNP: single nucleotide polymorphism
PFam: *Borrelia* protein family
IGS: rRNA intergenic sequence
