# Supplementary material for: Plasmid diversity and phylogenetic consistency in the Lyme disease agent Borrelia burgdorferi
Source: BMC Genomics. 2017 Feb 15;18:165. doi: 10.1186/s12864-017-3553-5 (PMC5310021; doi:10.1186/s12864-017-3553-5)
Supplement: Additional file 1: Figure S1. — B. burgdorferi plasmid PFam32 protein tree. Figure S2. Comparative maps of linear plasmids in 14 B. burgdorferi isolates. Maps of the following linear plasmids are shown in the following panels: A, lp5; B, lp17; C, lp21; D, lp25; E, lp28-1; F, lp28-2, lp28-6, lp28-7 and lp28-9; G, lp28-3; H, lp28-4; I, lp28-5; J, lp28-6; K, lp32-3; L, lp36; M, lp38; N, lp54; O, lp56. Figure S3. Deletions in cp32 circular plasmids. Figure S4. Comparative cp9 plasmid maps. Figure S5. All possible recombination products. Figure S6. Ancestral transfer of genetic material from the B. burgdorferi chromosome right end to a linear plasmid. Table S1. Linear plasmid locations of selected genes. (PDF 3869 kb) [file 12864_2017_3553_MOESM1_ESM.pdf]

Table of Contents - SUPPLEMENTARY MATERIAL for Casjens *et al.*

Figure	page
Figure S1. *Borrelia burgdorferi* plasmid PFam32 protein tree	2-3
Figure S2. Comparative maps of linear plasmids in fourteen *B. burgdorferi* isolates	4-19
Panel A, lp5	5
Panel B, lp17	6
Panel C, lp21	7
Panel D, lp25	8
Panel E, lp28-1	9
Panel F, lp28-2, lp28-7, lp28-9 and one lp28-6	10
Panel G, lp28-3	11
Panel H, lp28-4	12
Panel I, lp28-5	13
Panel J, lp28-6	14
Panel K, lp32-3	15
Panel L, lp36	16
Panel M, lp38	17
Panel N, lp54	18
Panel O, lp56	19
Figure S3. Deletions in cp32 circular plasmids	20
Figure S4. Comparative cp9 maps	21
Figure S5. All possible recombination products	22
Figure S6. Past transfer of genetic material from the *B. burgdorferi* chromosome right end to a linear plasmid	24
Table S1. Linear plasmid location of selected genes	25-26

Figure S1. *Borrelia burgdorferi* plasmid PFam32 protein tree.

PFAm32 amino acid sequences were aligned and an unrooted maximum likelihood tree was created by RAxML using the PROTGAMMAWAG model (Stamatakis A, RAxML version 8: a tool for phylogenetic analysis and post-analysis of large phylogenies. *Bioinformatics* 2014, 30:1312-1313); bootstrap values from 100 trials are shown for the nodes that define the PFam groups. A scale bar for number of amino acid substitutions per aligned site is shown at the left. At the tip of each branch the names of the plasmids that encode the PFam32 proteins are indicated in large bold text, and *Borrelia* isolates carrying them are indicated in smaller text ("Sh" indicates strain Sh-2-82; see text). The colored boxes highlight the new plasmid types discussed in this report. A neighbor-joining Clustal X (Larkin MA, Blackshields G, Brown NP, Chenna R, McGettigan PA, McWilliam H, Valentin F, Wallace IM, Wilm A, Lopez R *et al*. Clustal W and Clustal X version 2.0. *Bioinformatics* 2007, 23:2947-2948) tree gave a very similar and robust tree with identical groupings at the branch tips (not shown).

Figure S1

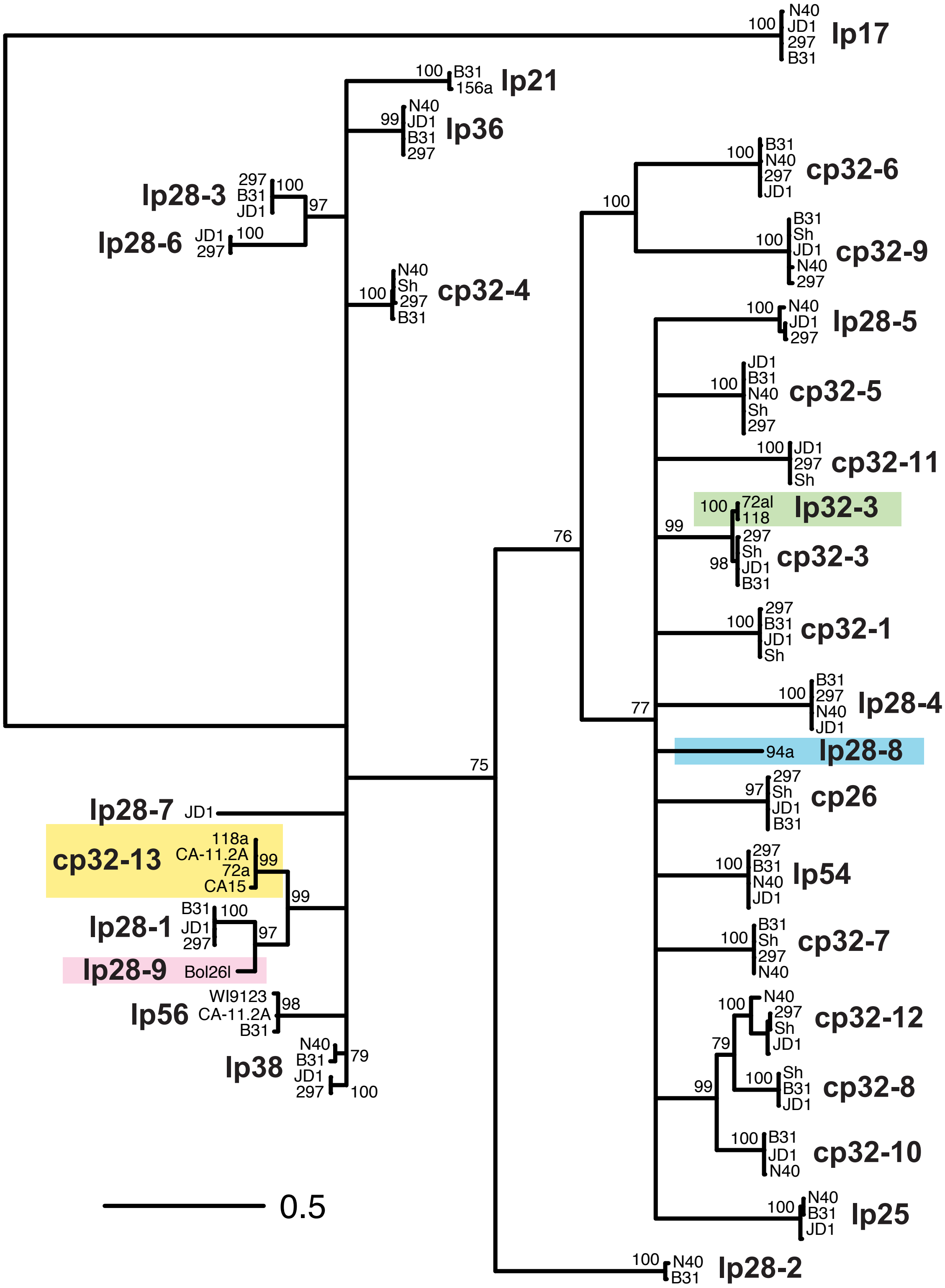
lp17
N40
JD1
297
B31
100
lp21
B31
156a
lp36
N40
JD1
B31
297
99
lp28-3
297
B31
JD1
100
97
lp28-6
JD1
297
cp32-4
N40
Sh
297
B31
cp32-6
B31
N40
297
JD1
cp32-9
B31
Sh
JD1
N40
297
lp28-5
N40
JD1
297
cp32-5
JD1
B31
N40
Sh
297
cp32-11
JD1
297
Sh
lp32-3
72al
118
cp32-3
297
Sh
JD1
B31
98
99
76
cp32-1
297
B31
JD1
Sh
lp28-4
B31
297
N40
JD1
77
lp28-8
94a
cp26
297
Sh
JD1
B31
97
75
lp28-7
JD1
cp32-13
118a
CA-11.2A
72a
CA15
lp28-1
B31
JD1
297
lp28-9
Bol26l
lp54
297
B31
N40
JD1
cp32-7
B31
Sh
297
N40
lp56
WI9123
CA-11.2A
B31
98
lp38
N40
B31
JD1
297
79
cp32-12
N40
297
Sh
JD1
cp32-8
Sh
B31
JD1
cp32-10
B31
JD1
N40
lp25
N40
B31
JD1
0.5
lp28-2
N40
B31

Figure S2. Comparative maps of linear plasmids in fourteen *B. burgdorferi* isolates.

Linear plasmid reading frame maps are shown with the six possible reading frames (top three rightward frames and bottom three leftward) with stop codons marked as vertical lines that span the frame rectangle, and potential start codons are indicated by short vertical lines. The maps were created with DNA Strider (Douglas SE: DNA Strider. A Macintosh program for handling protein and nucleic acid sequences. *Methods Mol Biol* 1994, 25:181-194). Plasmid names are shown at the top of each panel and strain names at the right. Above the maps some gene names are indicated and some protein family names are shown in boxes are shown in boxes (protein families defined by Casjens SR *et al*.: Genome stability of Lyme disease spirochetes: comparative genomics of *Borrelia burgdorferi* plasmids. *PLoS One* 2012, 7:e33280). Plasmid subtype Roman numerals are indicated at the left. Blue shading between maps indicates homologous regions and percent identity is indicated for selected regions on the shading. In each panel the same shading color on the maps indicates homologous regions.

Maps of the following linear plasmids are shown in the following panels: A, lp5; B, lp17; C, lp21; D, lp25; E, lp28-1; F, lp28-2, lp28-7, lp28-9 and one lp28-6; G, lp28-3; H, lp28-4; I, lp28-5; J, lp28-6; K, lp32-3; L, lp36; M, lp38; N, lp54; O, lp56.

Panel S2B

In panel S2B numbers in blue circles with black numbers mark the eleven novel sequence junctions present in the lp17 plasmids. Red "C", "D" and "E" above the maps indicate three indel alleles, and red numbers above the maps at about 11.6 kbp of the bottom scale are the number of repeats present in this tandem repeat array.

Figure S2A

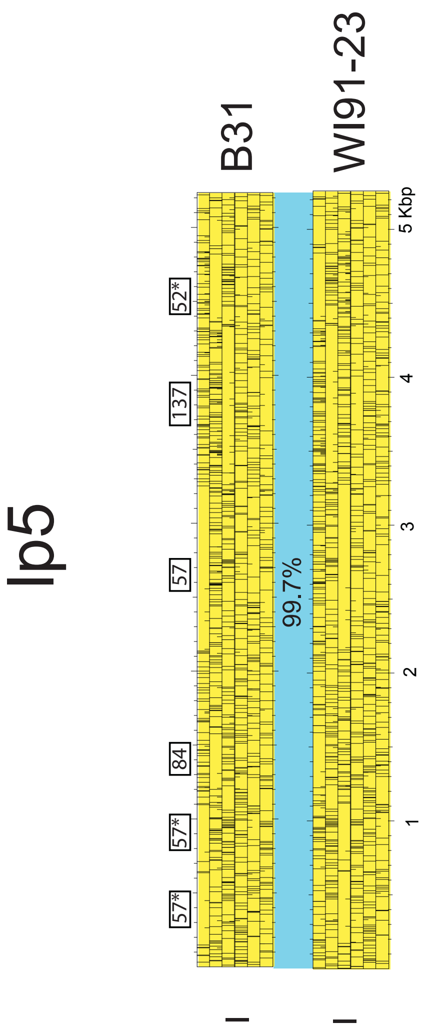

In panel S2B numbers in blue circles with black numbers mark the eleven novel sequence junctions present in the lp17 plasmids. The larger genes are colored red and large pseudogenes are colored green.

Figure S2B

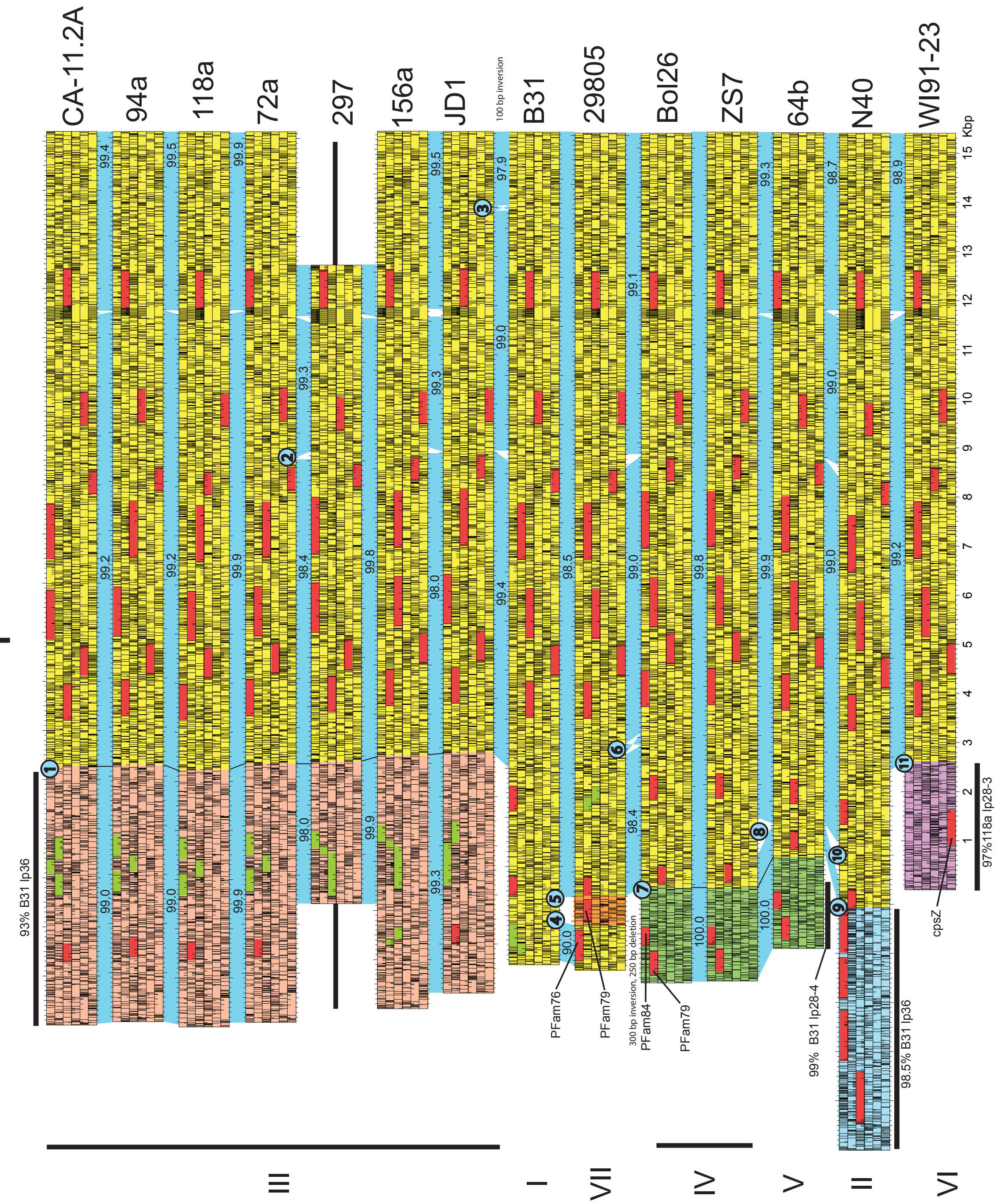

Figure S2C

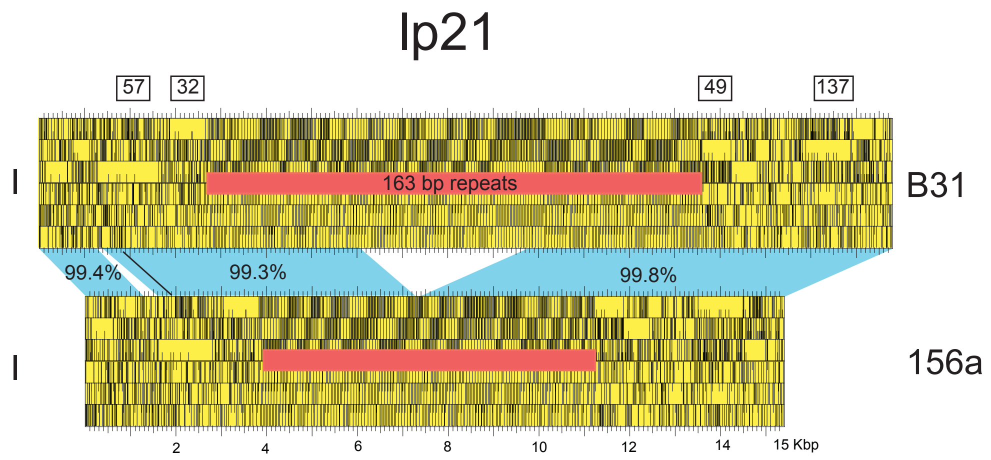
lp21
57
32
49
137
I
163 bp repeats
B31
99.4%
99.3%
99.8%
I
156a
2
4
6
8
10
12
14
15 Kbp

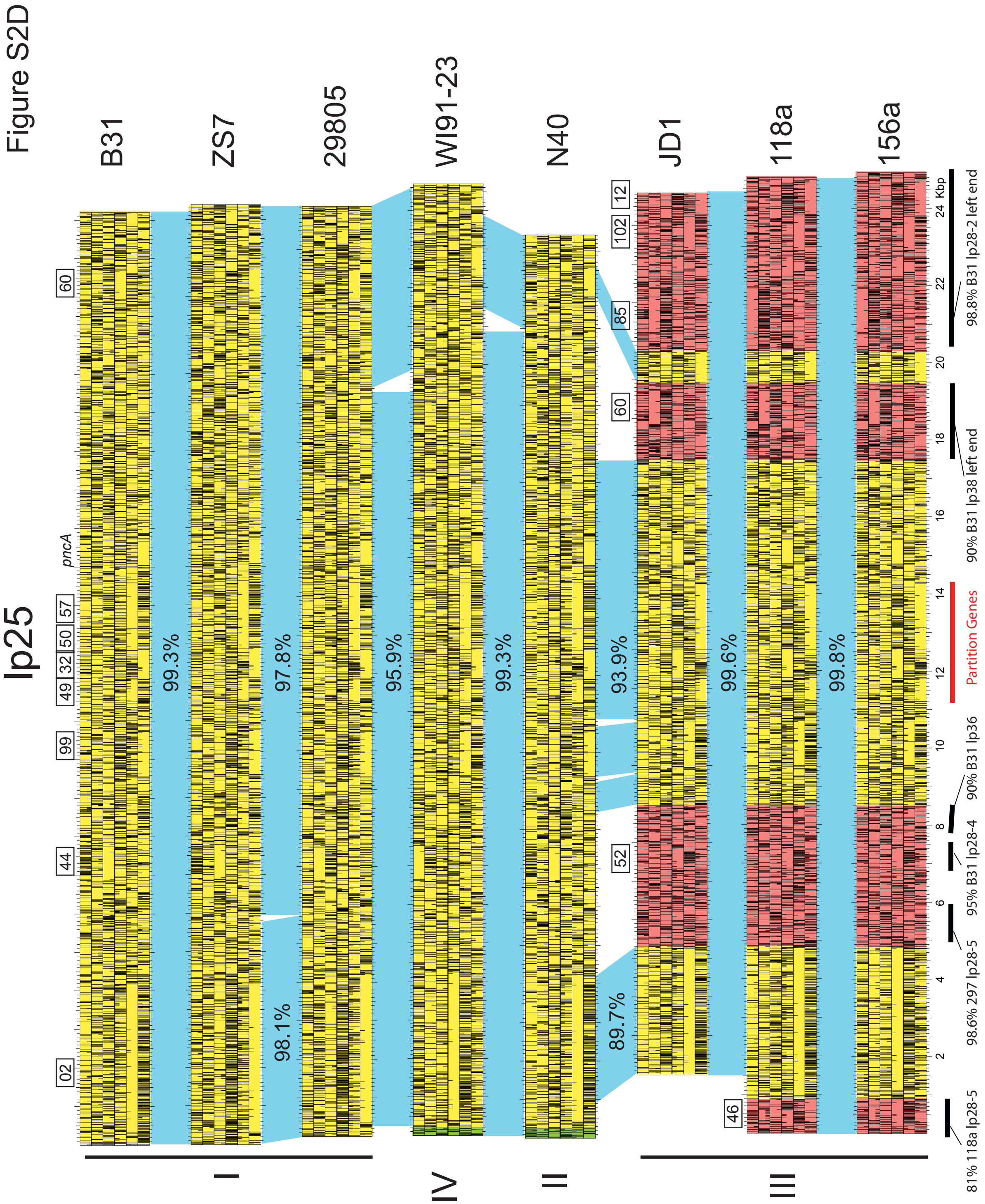
Figure S2D
lp25
B31
ZS7
29805
WI91-23
N40
JD1
118a
156a
99.3%
97.8%
98.1%
95.9%
99.3%
93.9%
89.7%
99.6%
99.8%
02
44
99
49
32
50
57
pncA
60
12
102
85
60
52
46
I
IV
II
III
Kbp
81% 118a lp28-5
98.6% 297 lp28-5
95% B31 lp28-4
90% B31 lp36
Partition Genes
90% B31 lp38 left end
98.8% B31 lp28-2 left end

Figure S2E

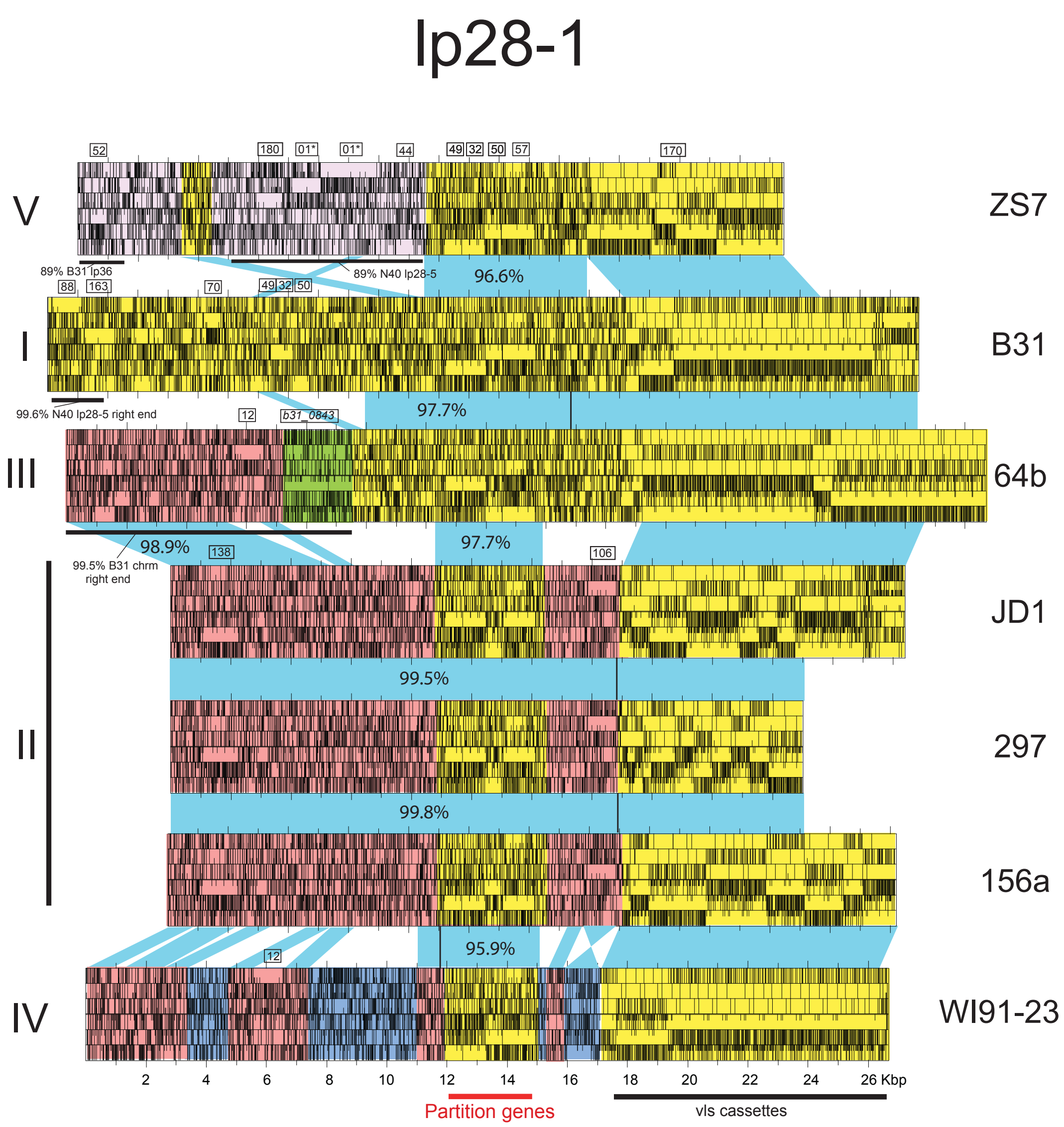
lp28-1
V
I
III
II
IV
ZS7
B31
64b
JD1
297
156a
WI91-23
89% B31 lp36
89% N40 lp28-5
96.6%
99.6% N40 lp28-5 right end
97.7%
b31_0843
98.9%
99.5% B31 chrm right end
97.7%
99.5%
99.8%
95.9%
2
4
6
8
10
12
14
16
18
20
22
24
26 Kbp
Partition genes
vls cassettes

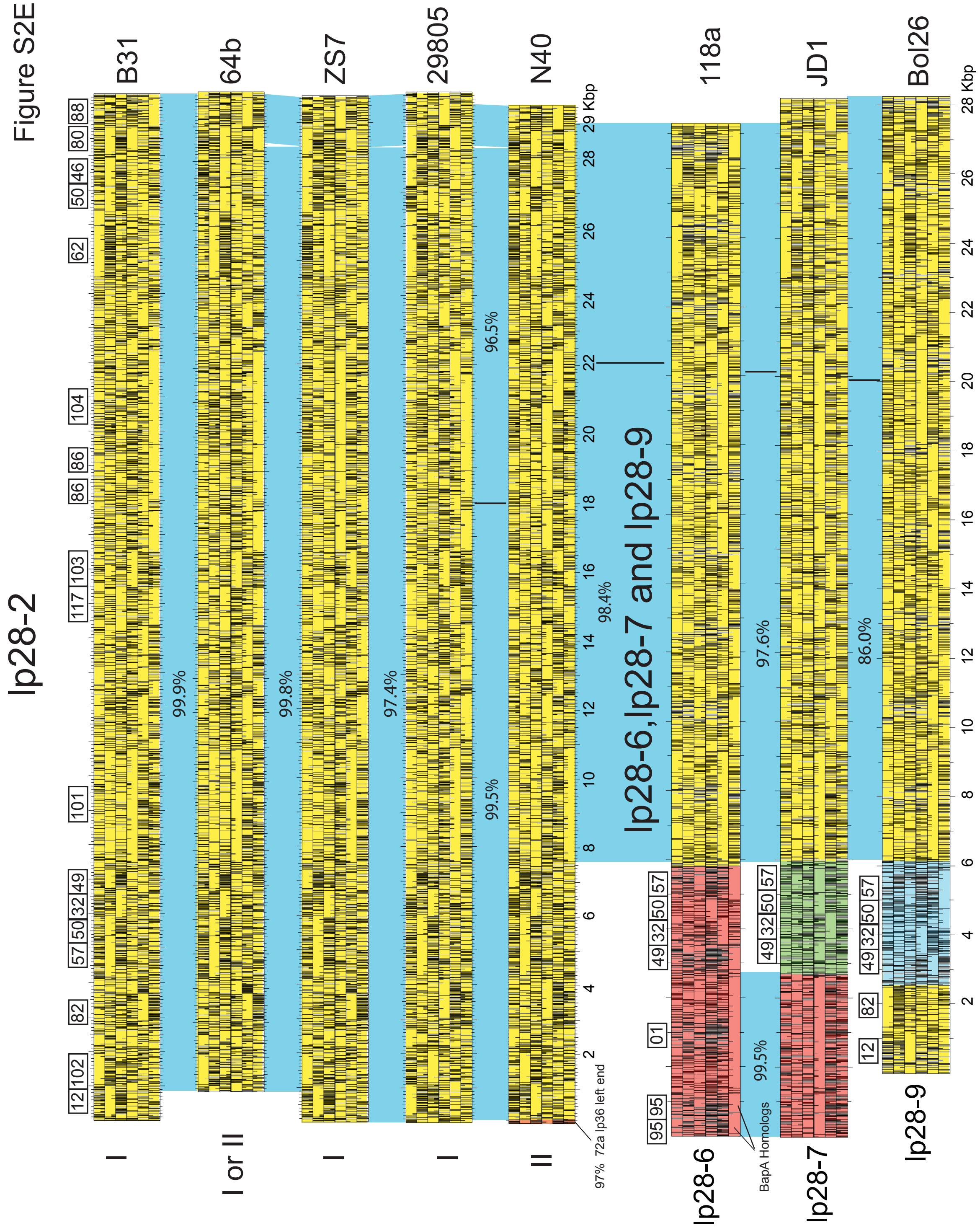
Figure S2E
lp28-2
B31
64b
ZS7
29805
N40
118a
JD1
Bol26
I
I or II
I
I
II
lp28-6,lp28-7 and lp28-9
lp28-6
lp28-7
lp28-9
99.9%
99.8%
97.4%
99.5%
96.5%
98.4%
97.6%
86.0%
99.5%
BapA Homologs
97% 72a lp36 left end
29 Kbp
28 Kbp

Figure S2G

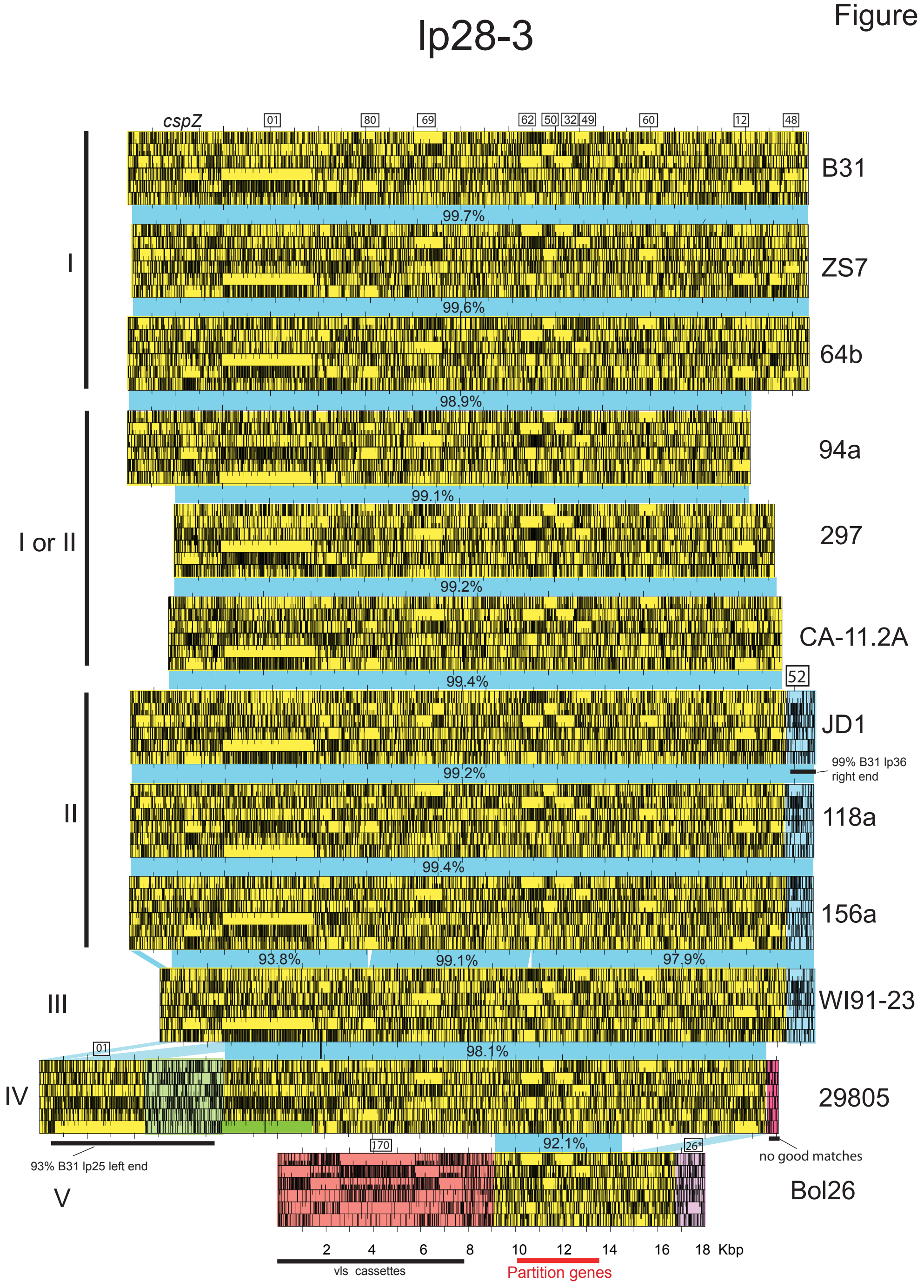
lp28-3
cspZ
01
80
69
62
50
32
49
60
12
48
B31
99.7%
ZS7
99.6%
64b
98.9%
94a
99.1%
297
99.2%
CA-11.2A
99.4%
52
JD1
99% B31 lp36 right end
99.2%
118a
99.4%
156a
93.8%
99.1%
97.9%
WI91-23
98.1%
29805
170
92.1%
26
no good matches
93% B31 lp25 left end
Bol26
I
I or II
II
III
IV
V
2
4
6
8
10
12
14
16
18 Kbp
vls cassettes
Partition genes

Figure S2H

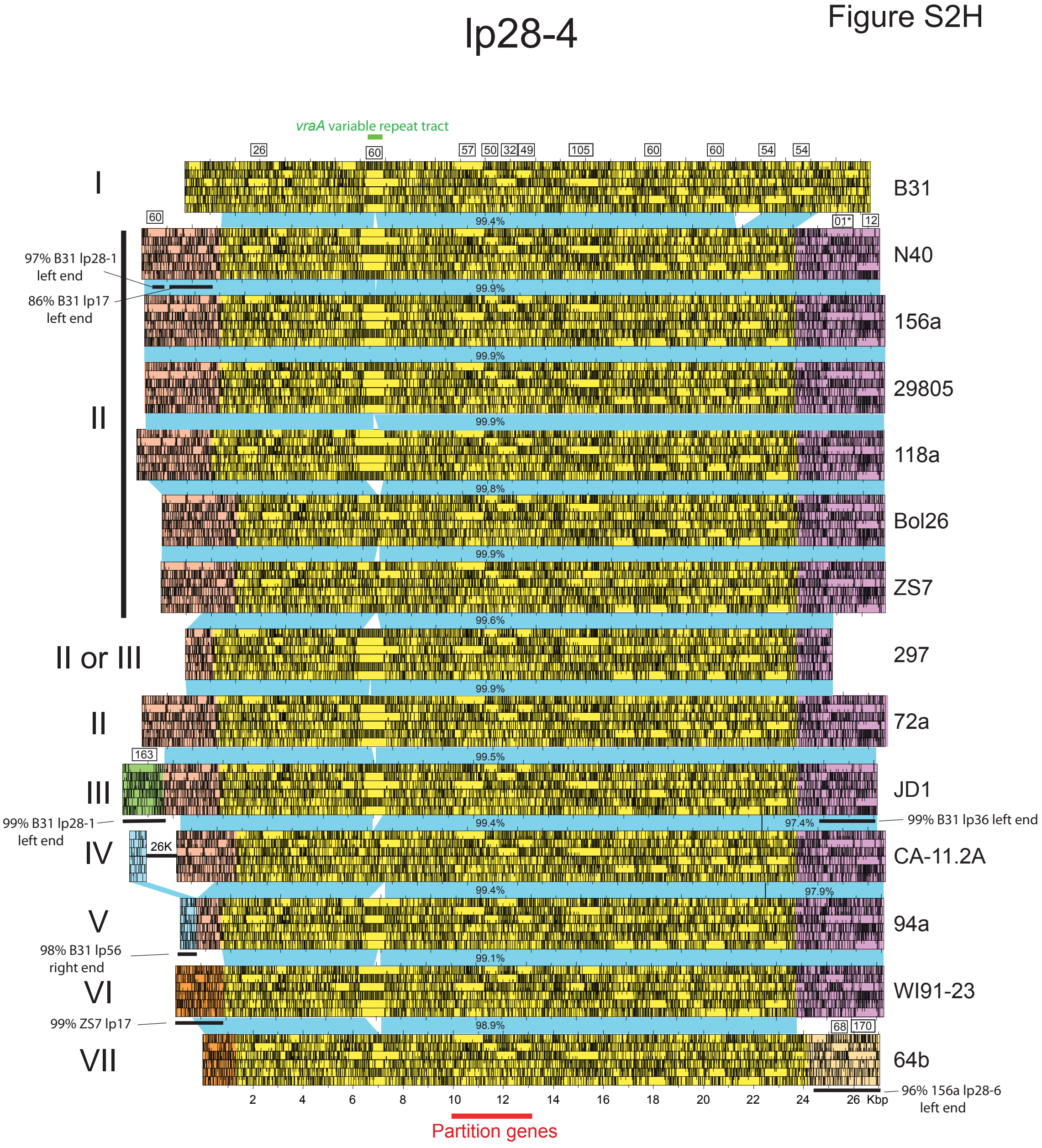

lp28-4
vraA variable repeat tract
I
II
II or III
III
IV
V
VI
VII
B31
N40
156a
29805
118a
Bol26
ZS7
297
72a
JD1
CA-11.2A
94a
WI91-23
64b
97% B31 lp28-1 left end
86% B31 lp17 left end
99% B31 lp28-1 left end
99% B31 lp36 left end
98% B31 lp56 right end
99% ZS7 lp17
96% 156a lp28-6 left end
26K
99.4%
99.9%
99.9%
99.9%
99.8%
99.9%
99.6%
99.9%
99.5%
99.4%
97.4%
99.4%
97.9%
99.1%
98.9%
2 4 6 8 10 12 14 16 18 20 22 24 26 Kbp
Partition genes

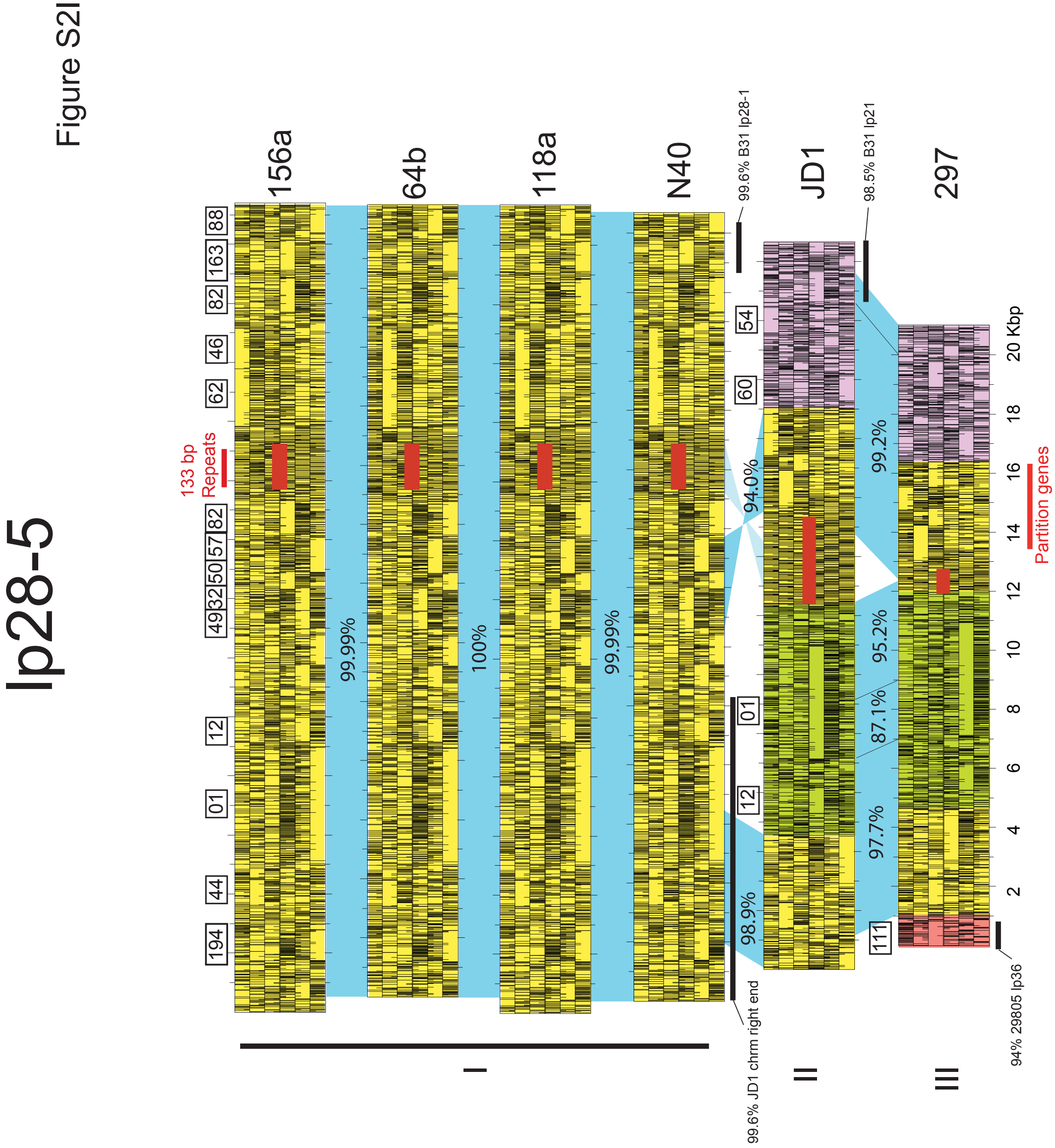
Figure S2I
lp28-5
156a
64b
118a
N40
JD1
297
99.99%
100%
99.99%
94.0%
98.9%
99.2%
95.2%
87.1%
97.7%
133 bp
Repeats
Partition genes
99.6% B31 lp28-1
98.5% B31 lp21
99.6% JD1 chrm right end
94% 29805 lp36
20 Kbp
18
16
14
12
10
8
6
4
2
88
163
82
46
62
82
57
50
32
49
12
01
44
194
54
60
01
12
111
I
II
III

lp28-6

Figure S2J

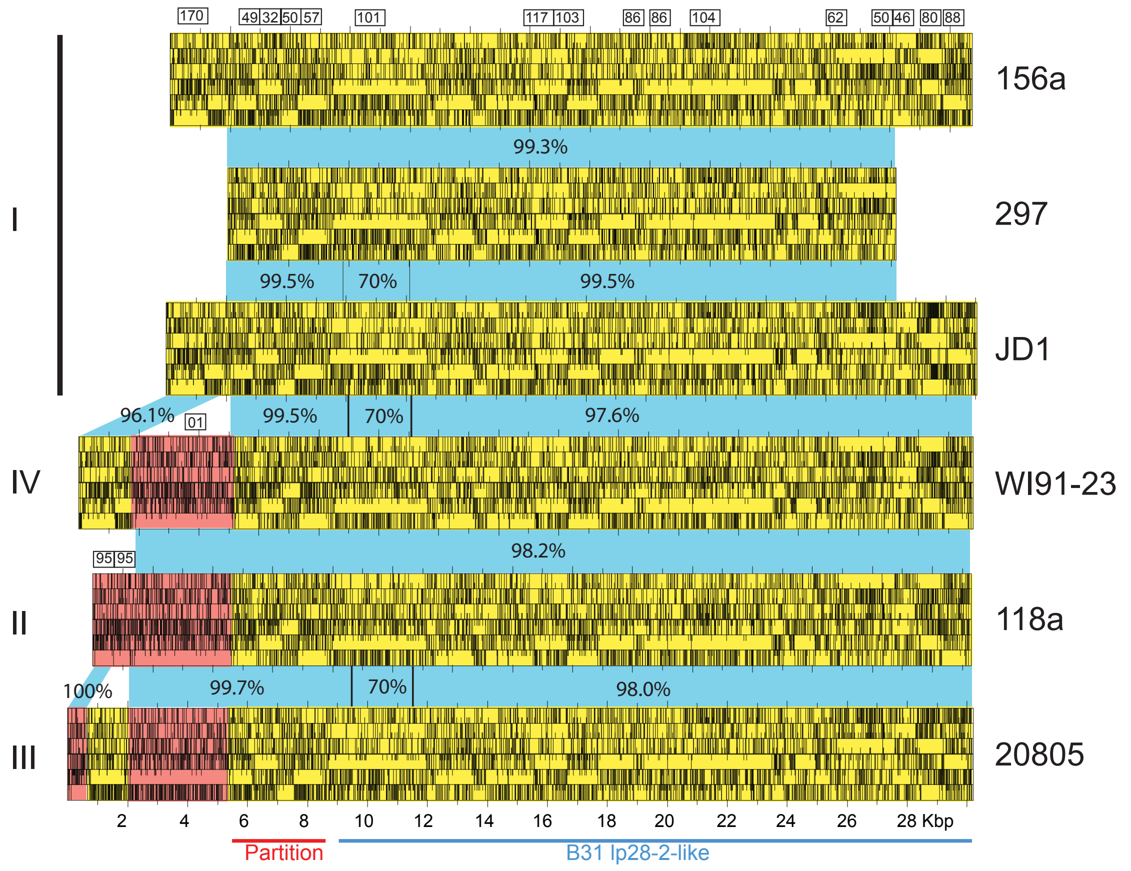

Figure S2K

lp32-3

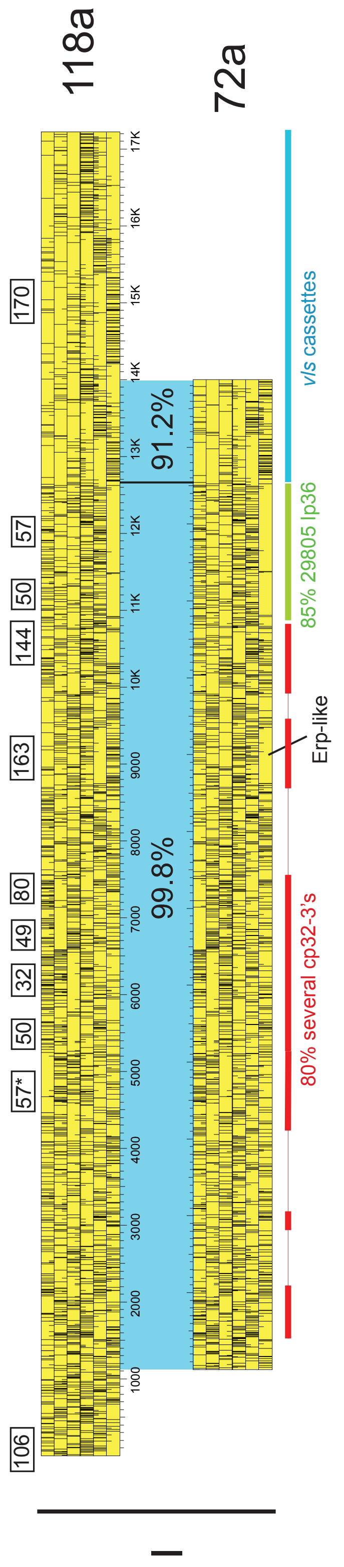

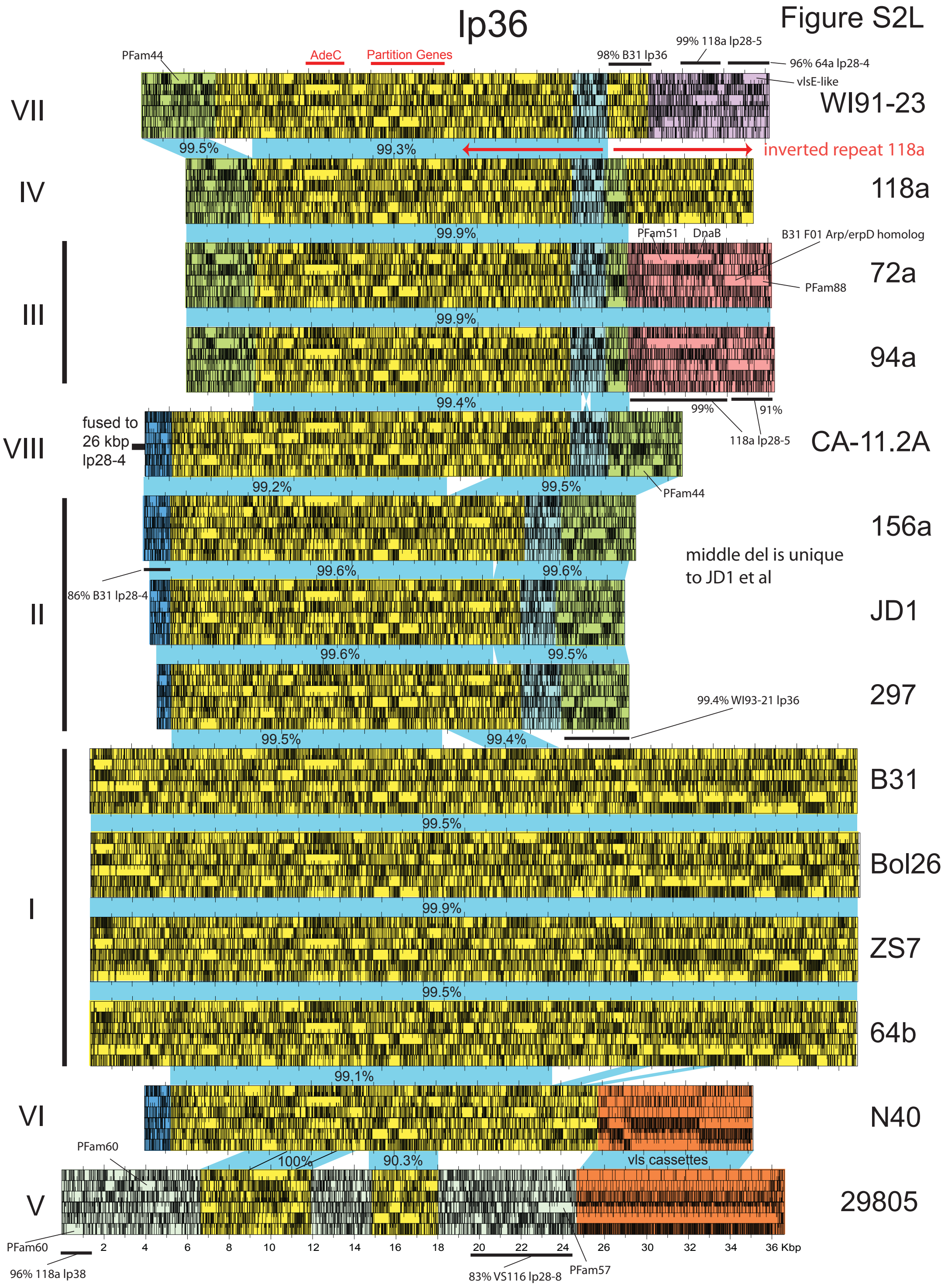
lp36
Figure S2L
PFam44
AdeC
Partition Genes
98% B31 lp36
99% 118a lp28-5
96% 64a lp28-4
vlsE-like
VII
WI91-23
99.5%
99.3%
inverted repeat 118a
IV
118a
99.9%
PFam51
DnaB
B31 F01 Arp/erpD homolog
72a
PFam88
III
99.9%
94a
99.4%
99%
91%
fused to
26 kbp
lp28-4
VIII
118a lp28-5
CA-11.2A
99.2%
99.5%
PFam44
156a
middle del is unique
to JD1 et al
99.6%
99.6%
86% B31 lp28-4
II
JD1
99.6%
99.5%
297
99.4% WI93-21 lp36
99.5%
99.4%
B31
99.5%
Bol26
I
99.9%
ZS7
99.5%
64b
99.1%
VI
N40
PFam60
100%
90.3%
vls cassettes
V
29805
PFam60
2 4 6 8 10 12 14 16 18 20 22 24 26 30 32 34 36 Kbp
96% 118a lp38
83% VS116 lp28-8
PFam57

Figure S2M

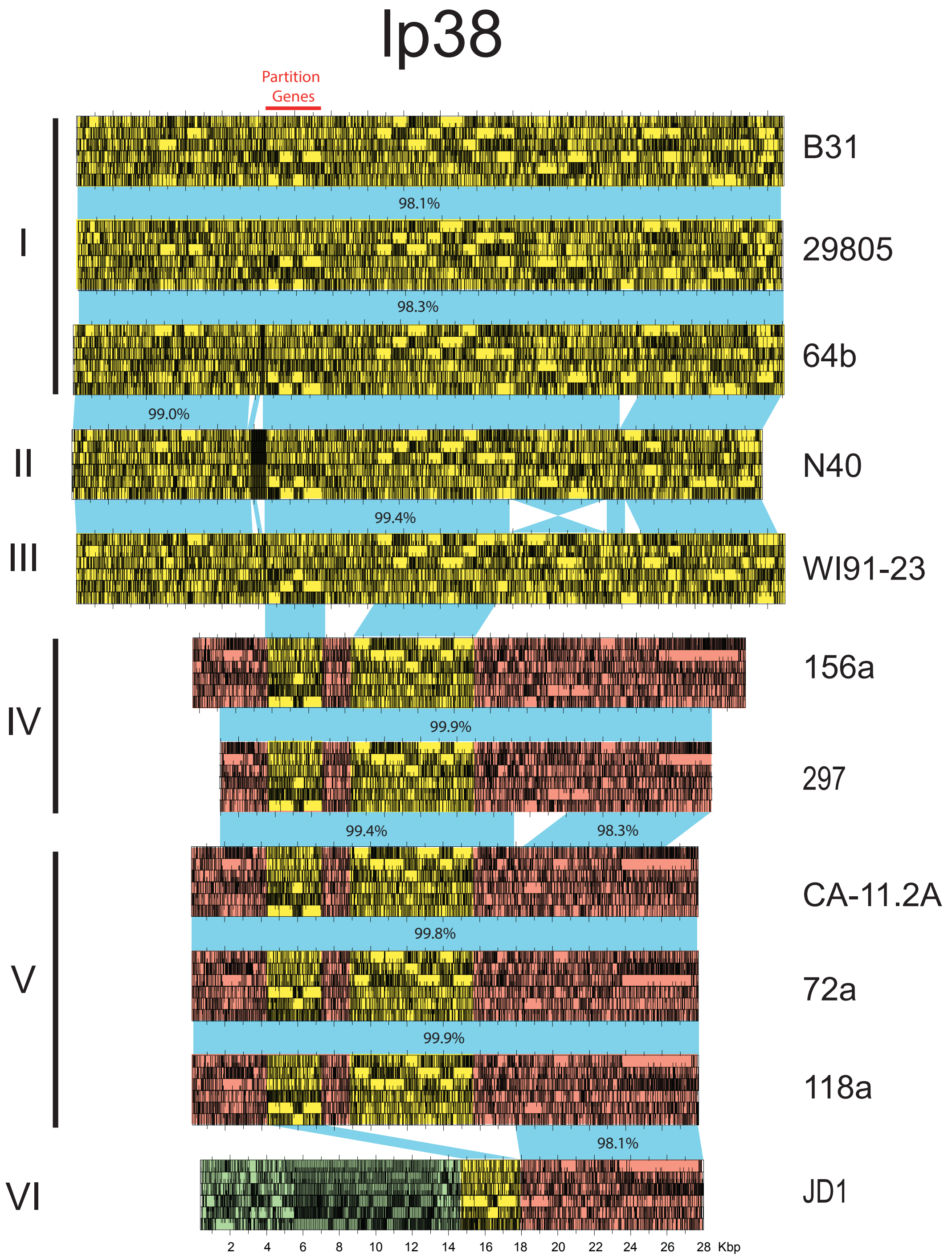
lp38
Partition Genes
B31
29805
64b
N40
WI91-23
156a
297
CA-11.2A
72a
118a
JD1
I
II
III
IV
V
VI
98.1%
98.3%
99.0%
99.4%
99.9%
99.4%
98.3%
99.8%
99.9%
98.1%
2
4
6
8
10
12
14
16
18
20
22
24
26
28 Kbp

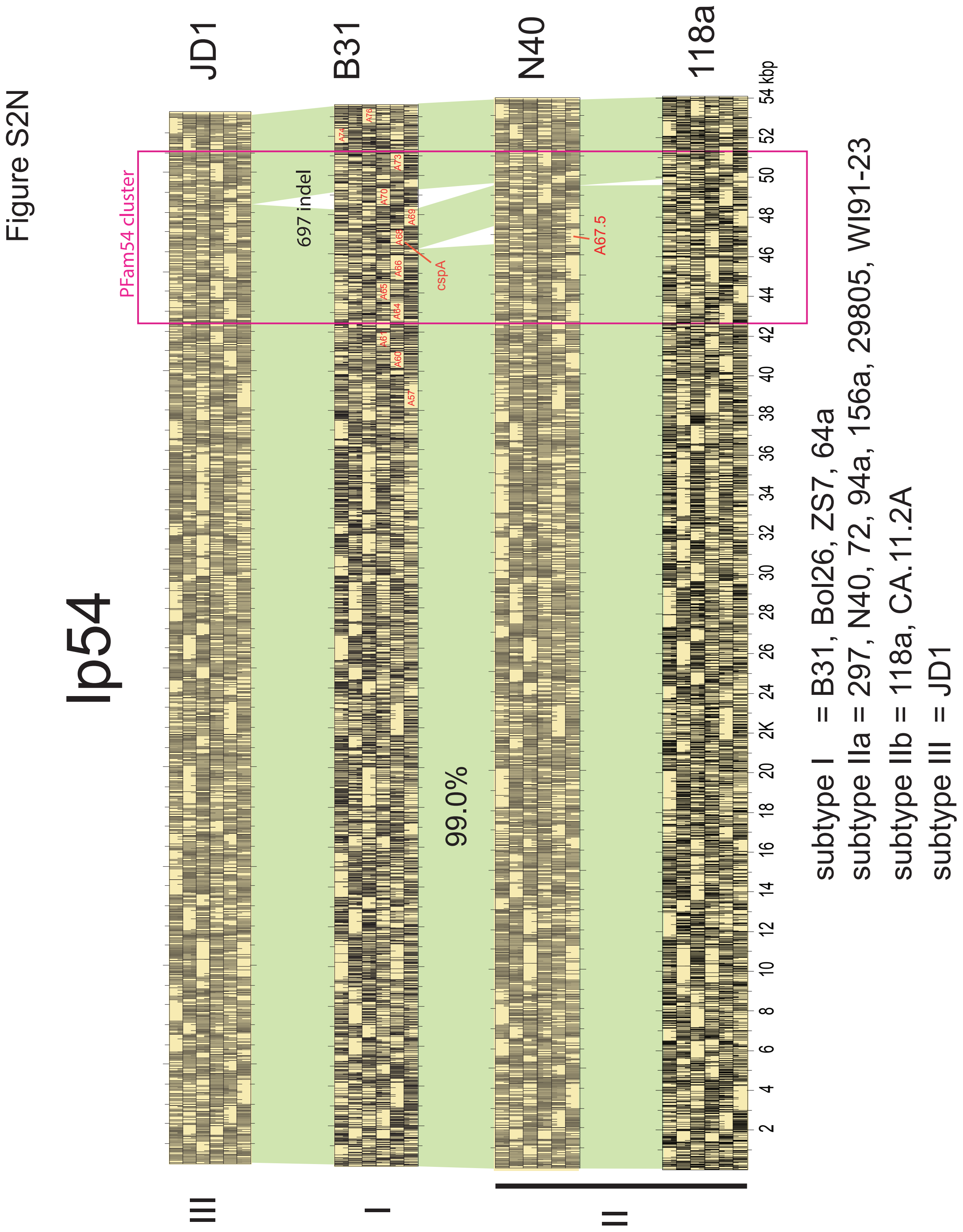
Figure S2N
lp54
JD1
B31
N40
118a
PFam54 cluster
697 indel
cspA
A67.5
99.0%
54 kbp
III
I
II
subtype I = B31, Bol26, ZS7, 64a
subtype IIa = 297, N40, 72, 94a, 156a, 29805, WI91-23
subtype IIb = 118a, CA.11.2A
subtype III = JD1

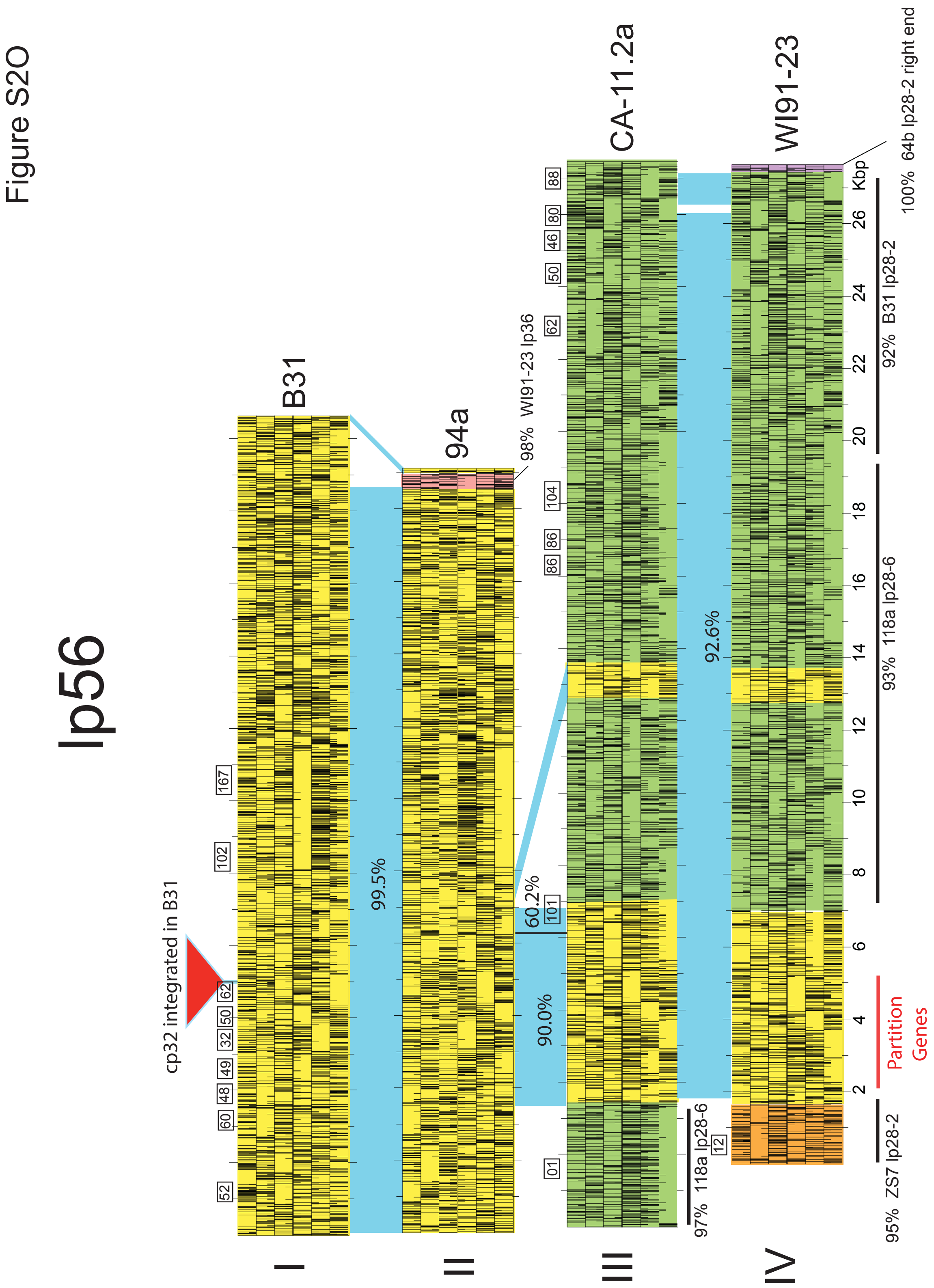
Figure S2O
lp56
cp32 integrated in B31
B31
94a
CA-11.2a
WI91-23
I
II
III
IV
99.5%
90.0%
60.2%
92.6%
98% WI91-23 lp36
97% 118a lp28-6
100% 64b lp28-2 right end
92% B31 lp28-2
93% 118a lp28-6
95% ZS7 lp28-2
Partition Genes
Kbp
52
60
48
49
32
50
62
102
167
01
101
86
86
104
62
50
46
80
88
12
2
4
6
8
10
12
14
16
18
20
22
24
26

Figure S3. Deletions in cp32 circular plasmids.

The long deletions in cp32 plasmids are indicated by thin lines with the deletion names (also see Figure 1 of text). A scale in kbp is shown below, and the plasmid sequences are oriented parallel to the previously reported strain B31 cp32 plasmids (Casjens S, *et al*: A bacterial genome in flux: the twelve linear and nine circular extrachromosomal DNAs in an infectious isolate of the Lyme disease spirochete *Borrelia burgdorferi*. *Mol Microbiol* 2000, 35:490-516).

Figure S3

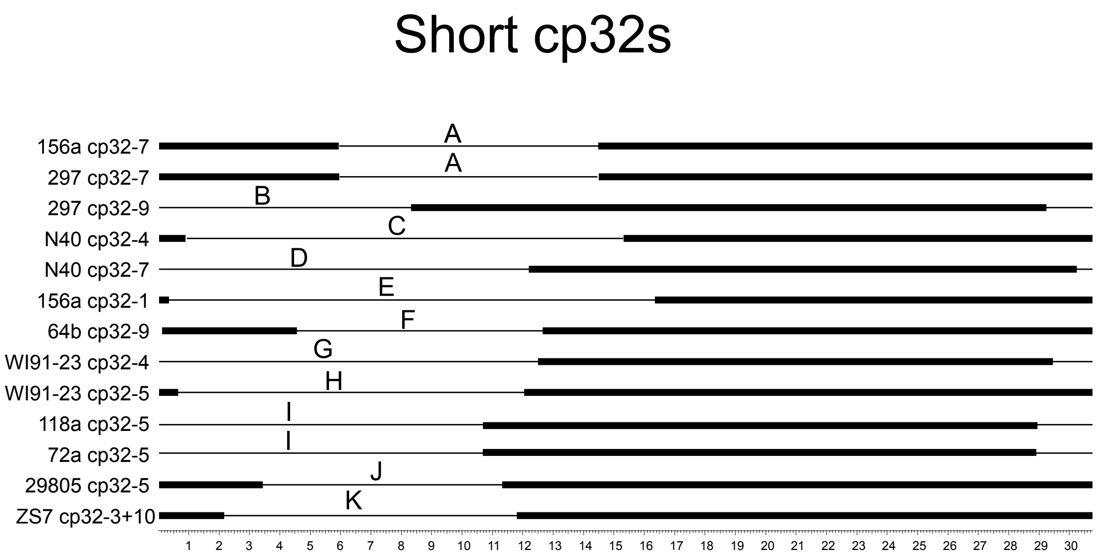

Figure S4. Comparative cp9 maps.

Cp9 plasmid maps are displayed as described in Supplementary figure S2. Plasmid WI91-23#B has remained in three contigs due to sequence assembly difficulties.

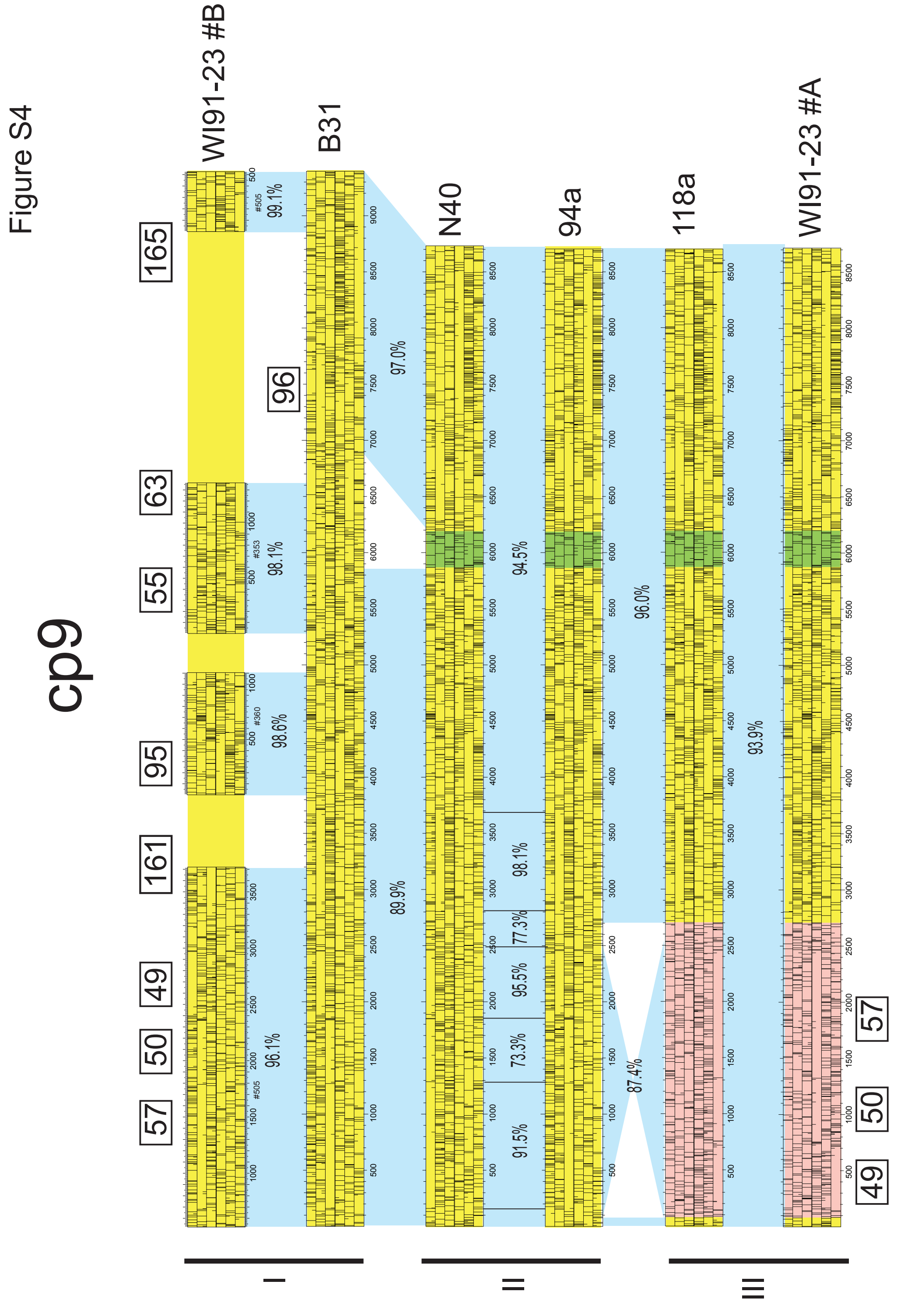

Figure S5. All possible recombination products.

The number of possible ways to randomly (non-homologously) recombine two identical sequences in a given orientation is equal to the number of bp squared, since each bp in one sequence can abut any of the bp in the other sequence. However, there are several possible orientations in which such an operation can be performed.

Panel A shows that two identical sequences represented by black and red arrows can be oriented head-to-head (above) or head-to-tail (below). Each of these can give rise to two sets of reciprocal products; in the figure each product represents all possible ways to recombine them in the orientations shown. If the sequences represented by the red and black arrows are identical, the bottom two sets of reciprocal products - "heads outward' and “tails outward” - are clearly different sequences. However, the top two product sets are identical since the red and black sequences are the same sequence. Thus there are three different sets of products, the number of which in each case is the number of bp in the sequence squared; hence, the total number of possible recombinants is = 3 x $\#bp^2$.

Panel B shows this by example. The four boxes list all possible ways in which two identical double-stranded four bp sequences can be joined together so that every recombinant has at least one bp from both parents. There are 16 (4 squared) ways to join any 4 bp sequence to another 4 bp sequence, and there are four possible orientations to join them shown in the four boxes (red and black sequences show the orientations that are recombined in each box). However, the recombined sequences in the upper-left and lower-right boxes are the reverse complements of one another when the two sequences are identical (the top strand in one is equivalent to the bottom strand in the other and *vice versa*). The recombined sequences in the upper-right and lower-left boxes are unique. Therefore, the number of possible different recombinant sequences is 3 x 4^2 p = 48 in this example.

Figure S5

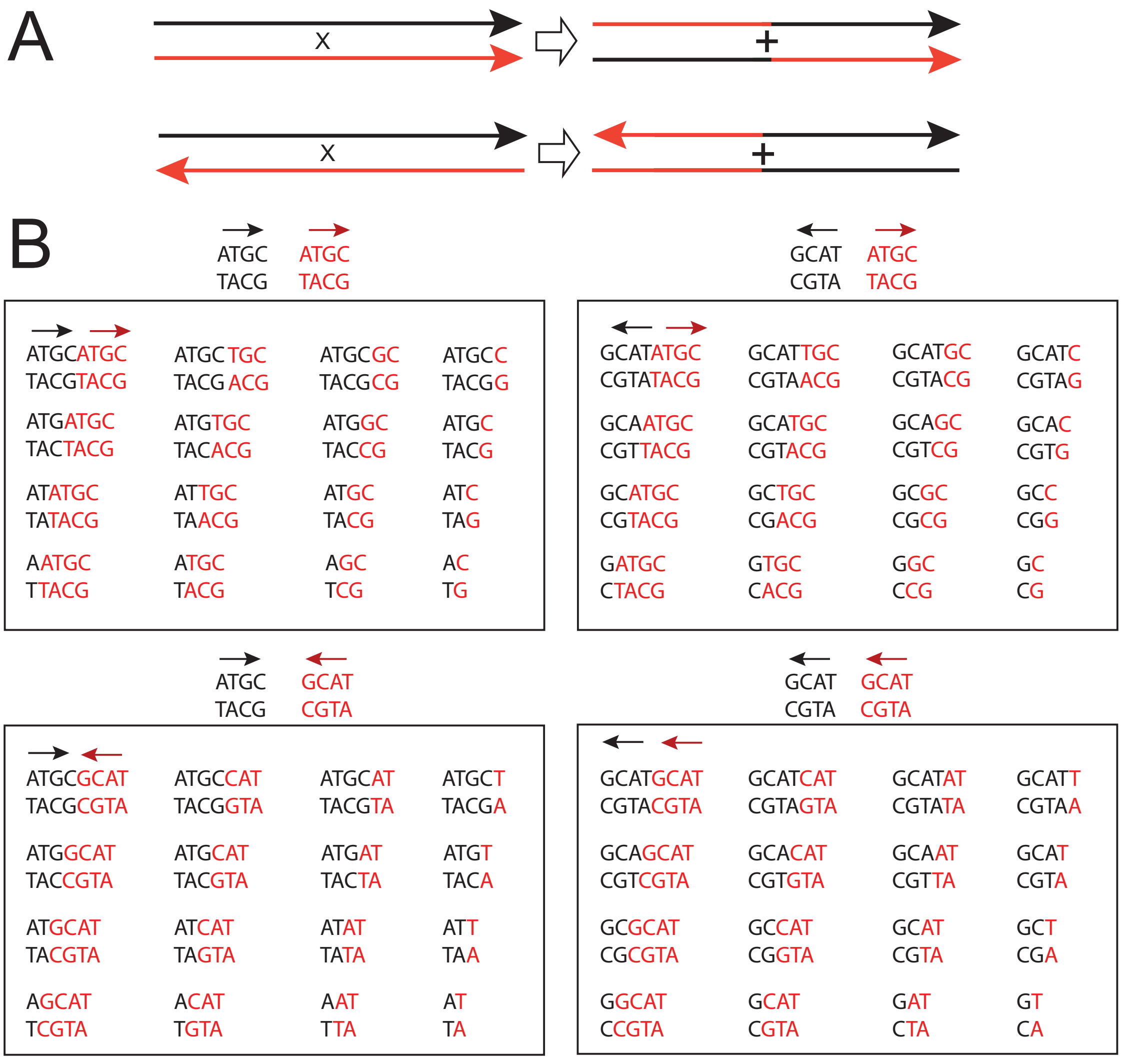
A
X
+
X
+
B
ATGC ATGC
TACG TACG
ATGCATGC ATGCTGC ATGCGC ATGCC
TACGTACG TACGACG TACGCG TACGG
ATGATGC ATGTGC ATGGC ATGC
TACTACG TACACG TACCG TACG
ATATGC ATTGC ATGC ATC
TATACG TAACG TACG TAG
AATGC ATGC AGC AC
TTACG TACG TCG TG
GCAT ATGC
CGTA TACG
GCATATGC GCATTGC GCATGC GCATC
CGTATACG CGTAACG CGTACG CGTAG
GCAATGC GCATGC GCAGC GCAC
CGTTACG CGTACG CGTCG CGTG
GCATGC GCTGC GCGC GCC
CGTACG CGACG CGCG CGG
GATGC GTGC GGC GC
CTACG CACG CCG CG
ATGC GCAT
TACG CGTA
ATGCGCAT ATGCCAT ATGCAT ATGCT
TACGCGTA TACGGTA TACGTA TACGA
ATGGCAT ATGCAT ATGAT ATGT
TACCGTA TACGTA TACTA TACA
ATGCAT ATCAT ATAT ATT
TACGTA TAGTA TATA TAA
AGCAT ACAT AAT AT
TCGTA TGTA TTA TA
GCAT GCAT
CGTA CGTA
GCATGCAT GCATCAT GCATAT GCATT
CGTACGTA CGTAGTA CGTATA CGTAA
GCAGCAT GCACAT GCAAT GCAT
CGTCGTA CGTGTA CGTTA CGTA
GCGCAT GCCAT GCAT GCT
CGCGTA CGGTA CGTA CGA
GGCAT GCAT GAT GT
CCGTA CGTA CTA CA

Figure S6. Past transfer of genetic material from the *B. burgdorferi* chromosome right end to a linear plasmid.

A dot matrix plot compares strain 64b linear plasmid lp28-1 to the right end of the strain B31 chromosome, and diagrammatic maps are shown at the top and left of these two DNAs, respectively. Map regions of the same color have very similar sequence and the same putative origin, and gene names are indicated on the map. The yellow plasmid region appears to have been obtained through recombination with a B31-like chromosome (see text). The plot was constructed by DNA Strider (Douglas SE: DNA Strider. A Macintosh program for handling protein and nucleic acid sequences. *Methods Mol Biol* 1994, 25:181-194).

Figure S6

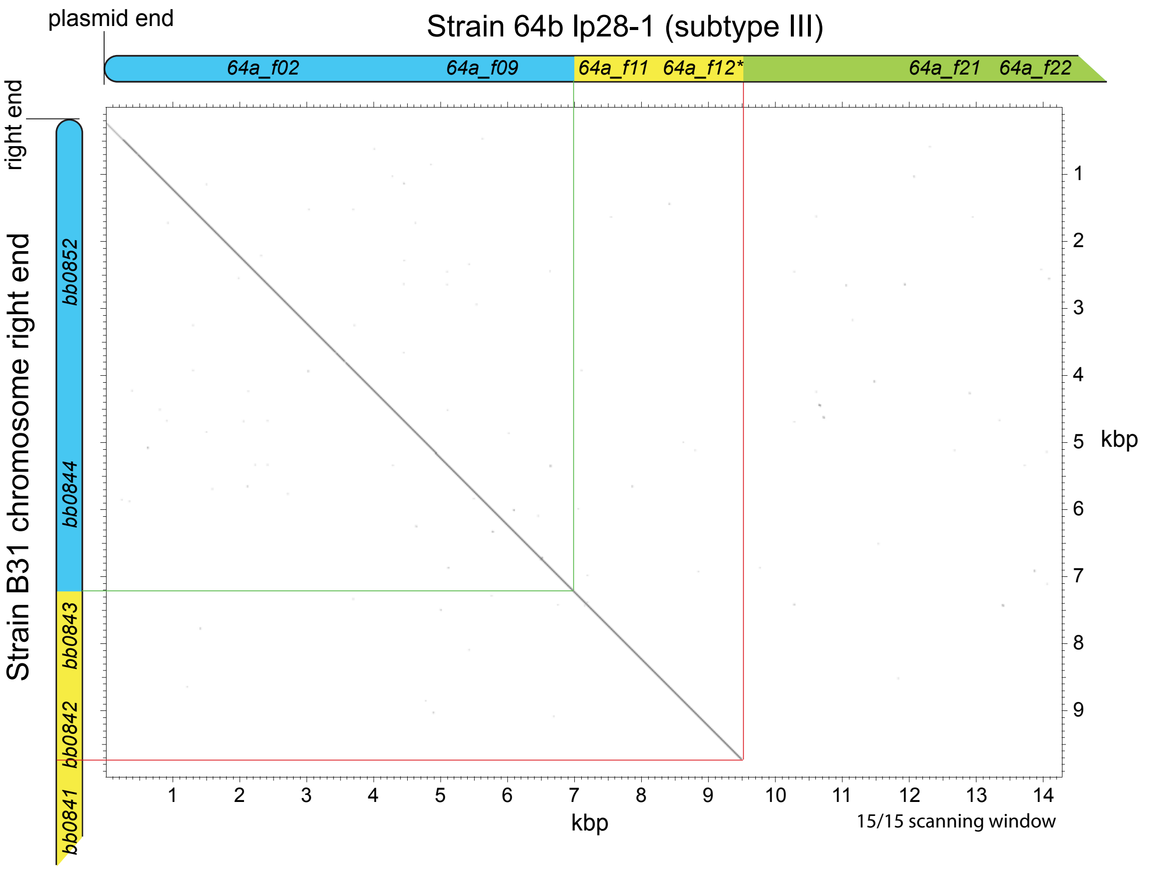

Table S1. Linear plasmid location of selected genes.

The linear plasmids that carry selected genes each of in the fourteen *B. burgdorferi* genome sequences are indicated in the table. Gene names used here are as follows: *adeC*, adenine deaminase; *arp*, arthritis related protein; *cpsZ*, complement regulatory protein Z; *dbpAB*, decorin binding proteins A and B; *fbp*, fibronectin protein; *plzB*, cyclic di-GMP binding protein; *hhaI*, HhaI-like restriction endonuclease; *ospAB*, outer surface proteins A and B; *ospD*, outer surface protein D; *pncA*, nicotinamidase; *res-mod1*, restriction-modification protein Fam01; *res-mod2*, restriction-modification protein Fam102/167; *vls*, variable outer surface protein cassettes.

Footnotes for Table S1

a. Gene is likely missing due to missing linear plasmid terminal sequence.

b. Relevant plasmid is known to have been originally present but was lost from the sequenced culture (see article text).

c. Lp54 gene content is quite constant so only a few examples this plasmid's many important genes are shown.

d. Gene is to be truncated relative to the longest paralogues; it may be non-functional.

e. Chromosomal single nucleotide polymorphism (SNP) type (Mongodin, E, *et al*.: Inter- and intra-specific pan-genomes of *Borrelia burgdorferi sensu lato*: Adaptive radiation and phylogenetic predictions. *BMC Genomics* 2013, 14:693).

f. lp28-4 and lp36 are apparently fused end-to-end in strain CA-11.2A; *fbp* is in the lp36-like portion.

Table S1

Linear plasmid locations of selected genes

	Borrelia burgdorferi sensu stricto													
gene (a)	B31	Bol26	ZS7	64b	N40	WI91- 23	29805	94a	72a	CA-11.2A	118a	297	156a	JD1
adeC	lp36	lp36	lp36	lp36	lp36	lp36	lp36	lp36	lp36	lp36	lp36	lp36	lp36	lp36
arp	lp28-1			lp28-5	lp28-5			lp36	lp28-4		lp28-5		lp28-5	lp28-4
cpsZ	lp28-3		lp28-3	lp28-3	(b)	lp17, lp28-3		lp28-3		lp28-3	lp28-3	lp28-3	lp28-3	lp28-3
dbpAB	lp54(c)	lp54	lp54	lp54	lp54	lp54	lp54	lp54	lp54	lp54	lp54	lp54	lp54	lp54
fbp	lp36	lp36	lp36	lp36	lp36	lp36		lp36	lp36	lp36 (f)	lp36	lp36	lp36	lp36
plzB			lp28-1	lp28-5	lp28-5						lp28-5		lp28-5	chrm
hhaI				lp28-5	lp28-5						lp28-5		lp28-5	
ospAB	lp54(c)	lp54	lp54	lp54	lp54	lp54	lp54	lp54	lp54	lp54	lp54	lp54	lp54	lp54
ospD	lp38			lp38	lp38	lp38	lp38							
pncA	lp25		lp25		lp25	lp25	lp25				lp25	(b)	lp25	lp25
res-mod1	lp25, lp28-3		lp25, lp28-3	lp28-3, lp28-5	lp25, lp28-5	lp25, lp28-3, lp28-6	lp25, lp28-3, lp28-3(d), lp28-6(d)	lp28-3, lp28-8	lp38	lp28-3, lp38, lp56(d)	lp25, lp28-3, lp28-5, lp28-6(d), lp38	lp28-3, lp28-5, lp38(d)	lp25, lp28-3, lp28-5, lp38	lp25(d), lp28-3, lp28-5, lp28-7(d), lp38, chromosome
res-mod2	lp56							lp56						
vls	lp28-1	lp28-3	lp28-1	lp28-1	lp36	lp28-1	lp36	lp28-8	lp32-3		lp32-3	lp28-1	lp28-1	lp28-1
Chrommosome SNP group (e)	A	A	A	A	B	B	B	C	C	C	C	D	D	D
